# Clinical classification in low back pain: best-evidence diagnostic rules based on systematic reviews

**DOI:** 10.1186/s12891-017-1549-6

**Published:** 2017-05-12

**Authors:** Tom Petersen, Mark Laslett, Carsten Juhl

**Affiliations:** 1 Back Center Copenhagen, Mimersgade 41, 2200 Copenhagen N, Denmark; 2 PhysioSouth Ltd, 7 Baltimore Green, Shirley, Christchurch, 8061 New Zealand; 3 Southern Musculoskeletal Seminars, Christchurch, New Zealand; 4 Research Unit for Musculoskeletal Function and Physiotherapy, Department of Sports Science and Clinical Biomechanics, University of Southern Denmark, Odense, Denmark; 5 Department of Rehabilitation, University Hospital of Copenhagen, Herlev and Gentofte, Niels Andersen Vej 65, 2900 Hellerup, Denmark

**Keywords:** Diagnostic accuracy, Sensitivity and specificity, Clinical examination, Low back pain classification, Clinical decision making

## Abstract

**Background:**

Clinical examination findings are used in primary care to give an initial diagnosis to patients with low back pain and related leg symptoms. The purpose of this study was to develop best evidence Clinical Diagnostic Rules (CDR] for the identification of the most common patho-anatomical disorders in the lumbar spine; i.e. intervertebral discs, sacroiliac joints, facet joints, bone, muscles, nerve roots, muscles, peripheral nerve tissue, and central nervous system sensitization.

**Methods:**

A sensitive electronic search strategy using MEDLINE, EMBASE and CINAHL databases was combined with hand searching and citation tracking to identify eligible studies. Criteria for inclusion were: persons with low back pain with or without related leg symptoms, history or physical examination findings suitable for use in primary care, comparison with acceptable reference standards, and statistical reporting permitting calculation of diagnostic value. Quality assessments were made independently by two reviewers using the Quality Assessment of Diagnostic Accuracy Studies tool. Clinical examination findings that were investigated by at least two studies were included and results that met our predefined threshold of positive likelihood ratio ≥ 2 or negative likelihood ratio ≤ 0.5 were considered for the CDR.

**Results:**

Sixty-four studies satisfied our eligible criteria. We were able to construct promising CDRs for symptomatic intervertebral disc, sacroiliac joint, spondylolisthesis, disc herniation with nerve root involvement, and spinal stenosis. Single clinical test appear not to be as useful as clusters of tests that are more closely in line with clinical decision making.

**Conclusions:**

This is the first comprehensive systematic review of diagnostic accuracy studies that evaluate clinical examination findings for their ability to identify the most common patho-anatomical disorders in the lumbar spine. In some diagnostic categories we have sufficient evidence to recommend a CDR. In others, we have only preliminary evidence that needs testing in future studies. Most findings were tested in secondary or tertiary care. Thus, the accuracy of the findings in a primary care setting has yet to be confirmed.

**Electronic supplementary material:**

The online version of this article (doi:10.1186/s12891-017-1549-6) contains supplementary material, which is available to authorized users.

## Background

Identifying diagnostic, prognostic and treatment orientated subgroups of patients with low back pain (LBP] has been on the research agenda for many years [1, 2]. Diagnostic reasoning with a structural/pathoanatomical focus is common among clinicians [3], and it is regarded as an essential component of the biopsychosocial model [4–6]. Within this model, emphasis has been on the role of psychosocial considerations and how these factors can interfere with recovery. Indeed, there is good quality evidence for the predictive value of a set of psychosocial factors for poorer outcome in patients with LBP [7, 8]. These factors are multifactorial, interrelated, and only weakly associated to the development and prognosis of LBP [9], which might be one of the explanations why effects of treatments targeting those risk factors has been reported to be small, mostly short term, and there was little evidence that psychosocial treatments were superior to other active treatments [7, 10].

Maybe it is time to swing the pendulum towards the “bio” in the biopsychosocial model. There are many examples in medicine where the pathology has been identified prior to any effective treatments being developed making it an ongoing challenge to generate new diagnostic knowledge on which to base more effective treatment strategies in the future. Alongside clinicians, many researchers within the field of LBP feel that choosing the most effective treatment for the individual patient is not possible without better understanding of the biological component of the biopsychosocial model [4].

In 2003 the present authors suggested a diagnostic LBP classification system based on a review of the literature [11, 12]. This system has been fully or partly used in prognostic and outcome studies by other research groups [13–15]. The present study is driven by the obvious need for an update based on recent evidence. The relevance of an updated diagnostic classification is as follows:

First, diagnostic patterns of signs and symptoms from history and physical examination may assist the clinician in explaining the origin of pain to the patient and in directing treatment at the painful structure. Patients with persistent LBP often have misconceptions about what is going on [16], and may have been given all sorts of speculative explanations for their symptoms resulting in anxiety and confusion. These patients often seek an explanation about what is wrong [17], and new evidence suggests that offering clear explanations and information about aetiology, prognosis and interventions may improve patient outcomes [7]. Giving an explanation based on best evidence may contribute to 1) reducing the patient’s confusion and conceptual chaos, 2) reassurance that the clinician knows what is going on, 3) visualizing the potential benefit of treatment directed at the painful structure (mental imagery has been suggested to have potential in pain management [18, 19], 4) provided that the above efforts are successful, motivating the patient to open a therapeutic window.

Second, the need for studies testing the effect of treatment strategies for subgroups of patients with LBP in primary care has been emphasized in consensus-papers [1, 20] as well as current European guidelines [21]. Targeting treatment to classifications merely based on prognostic patient characteristics has not been convincingly successful in finding treatment modalities that are more beneficial than others [22]. A diagnostic classification may assist in generating hypotheses as to which treatment modalities are more likely to target the pain source for future testing in randomized trials.

Finally, an evidence-based clinical diagnosis with acceptable accuracy will reduce the need for invasive or expensive diagnostic methods (often with substantial waiting time and expense).

The focus of this review is to outline the diagnostic value of signs and symptoms for use in primary care without access to confirmatory paraclinical methods. The clinician must not mislead the patient, so it is important to distinguish between diagnostic labels that can be given to patients with reasonable confidence and those only suggesting suspected best evidence patho-anatomy. Therefore, it is of interest to identify signs and symptoms with the potential to diagnose common sources and causes of LBP i.e. intervertebral discs, sacroiliac joints, facet joints, bones, nerve roots, muscles, peripheral nerve tissue, and central nervous system sensitization.

Throughout this review, we use the term Clinical Diagnostic Rule (CDR) meaning that we have applied a clinical decision rule to the field of clinical diagnostics. A clinical decision rule “is a clinical tool that quantifies the individual contributions that various components of the history, physical examination, and basic laboratory results make toward the diagnosis, prognosis, or likely response to treatment in a patient. Clinical decision rules attempt to formally test, simplify, and increase the accuracy of clinicians’ diagnostic and prognostic assessments” [23].

The aim of this paper was to develop multi-faceted Clinical Diagnostic Rules (CDRs) for the lumbar spine using individual diagnostic accuracy scores based on best evidence for use in primary care clinical practice and research. If possible, single clinical examination findings would be clustered in CDRs based on well-defined criteria.

## Methods

The reporting of this review was based on the Preferred Reporting Items for Systematic reviews and Meta-analyses statement (PRISMA) [24].

### Eligibility criteria and study selection

To be included studies were required to meet the following criteria:

1. Participants had LBP with or without leg pain
2. Use of an appropriate reference standard as listed in Table 1.

**Table 1:** Reference standards for painful lumbosacral spine structures

Structure	Reference standard	References
Intervertebral disc	Provocation discography with control disc verification	[171]
Facet joint	Double block procedure in joint space or at nerve supply	[148]
Sacroiliac joint	Double block procedure in joint space	[172]
Nerve root involvement	Magnetic resonance imaging, myelography, or surgical findings with or without clinical findings	[173]
Spinal stenosis	Expert opinion based on radiographs, magnetic resonance imaging or surgical findings with or without clinical findings	[75, 174]
Spondylolisthesis	Sagittal plane rotation or translation movement on functional radiograph or translation on static radiograph	[152, 155]
Fracture	Radiographs, computed tomography or magnetic resonance imaging	[155]
Myofascial structures	None available.	
Peripheral nerve	None available.	
Central sensitization	Expert consensus
3. Evaluation of at least one clinical finding available to primary care clinicians.
4. Presentation of data enabling calculation of sensitivity and specificity.

For some diagnostic categories, recent systematic reviews were found covering our topic. These were included if they complied with the principles recommended by the Cochrane Collaboration [25]. In other categories, where searches in included systematic reviews were terminated before 2011, our searches were performed up to May 2015 from the date where the search of those reviews was terminated. In categories where no systematic reviews were found, we conducted systematic searches in the electronic databases PubMed, Embase, and CINAHL. Details of the search strategy are presented in Additional files 1, 2, 3 and 4. One of the authors (TP) reviewed the search results from the databases (titles and abstracts). Any titles and abstracts from studies that appeared to compare the results of clinical examination findings on patients with LBP with those of diagnostic reference standards were selected for full text review. Reference lists of selected studies were reviewed for additional studies. If necessary, authors were contacted for clarification of unclear reporting. The data extraction from the selected studies was prepared by one author (TP) and the second author (ML) reviewed the complete data extraction form for accuracy. Any disagreements were resolved by discussion. In diagnostic findings where no studies presenting sensitivity and specificity were found, studies presenting predictive values (sensitivity only) were included. We extracted values of diagnostic accuracy for clinical examination findings that were investigated by at least two studies.

### Reference standards

In this review, we used the best available reference standards for diagnosis of the relevant source and cause of LBP. See Table 1. Index tests results were reported if they were investigated in at least two studies using the best available reference standard.

### Quality assessment

Original studies were retrieved in full text and independently scored for quality and risk of bias using Quality Assessment of Diagnostic Accuracy Studies (QUADAS) in accordance with the recommendations of the Cochrane Handbook for Systematic Reviews of DTA [26]. Any disagreements were resolved by discussion. In a few cases, one of the present authors were co-authoring a paper or we were not able to acquire the original papers included in previous reviews. In these cases the results of QUADAS were transferred from the review in question to the present paper.

### Grading of recommendations

There is currently no consensus regarding criteria to assess the quality of evidence of diagnostic tests [27]. In this study, diagnostic values that were in agreement in more than two thirds of studies were included in our final recommendations. Downgrading of recommendations from strong to weak was made in cases with serious risk of bias due to verification bias, partial verification bias, differential verification, incorporation bias, or test review bias.

### Diagnostic accuracy measures

In order to be clinically useful, we considered the cut-off for a clinical finding to rule in the disorder to be a positive likelihood ratio (LR) above 2.0 [28], meaning that a positive index test will at least double the ratio of having the disorder compared to not having the disorder. This means that if the pretest probability is 0.3, the pretest odds is 0.3/0.7 = 0.43 and if the LR is 2.0 the posttest odds is 2*0.43 = 0.86 and the posttest probability can then be estimated to 0.46. For a useful clinical finding to rule out the disorder, we considered the cut-off to be a negative LR below 0.5 [28], meaning that a negative index test will reduce the odds of having the disorder at least by half compared to not having the disorder. Overall, the change from pretest to posttest chance of having the disorder in question depends on the pretest probability.

In summary, clinical examination findings that were investigated by at least two studies were included. Diagnostic values that were in agreement in more than two thirds of studies and met our predefined threshold of positive likelihood ratio ≥ 2 or negative likelihood ratio ≤ 0.5 were considered for the CDR.

### Statistics

A meta-analysis was considered if evidence of clinical homogeneity could be established. Clinical heterogeneity was assessed by comparing the similarity of patient samples, performance of tests, and reference standards. However, a qualitative synthesis of studies according to principles of best-evidence synthesis [29] was performed if studies were clinically heterogeneous.

## Results

Table 2 outlines the findings in each of the diagnostic categories that are supported by more than one study. Characteristics of the included studies are presented in Additional file 5. Results of the quality assessments are presented in Additional file 6. Results of the searches of the literature are presented in Additional files 7, 8, 9, 10, 1, 2, 3 and 4.

**Table 2:** Diagnostic accuracy of clinical tests for lumbar diagnoses that are investigated by more than one study

Structure	Sensitivity (95% CI)	Specificity (95% CI)	Positive LR (95% CI)	Negative LR (95% CI)
Intervertebral disc				
Studies supporting a diagnostic rule				
Centralization (P) Donelson 1997 [31]	0.64 (0.46–0.79)	0.70 (0.50–0.86)	2.1 (1.2–3.9)	0.52 (0.32–0.86)
Centralization (P)^a^ Young 2003 [32]	0.47 (0.22–0.73)	0.95 (0.62–1.0)	9.4 (0.6–146.9)	0.56 (0.35–0.91)
Centralization (P)^a^ Laslett 2005 [33]	0.40 (0.28–0.54)	0.94 (0.73–0.99)	6.9 (1.0–47.3)	0.63 (0.49–0.82)
Studies not supporting a diagnostic rule				
None				
Additional findings reported by more than one study				
Pain crosses midline^a^ (H) Young 2003 [32]	0.27 (0.11–0.52)	0.38 (0.14–0.69)	0.4 (0.2–1.2)	1.96 (0.76–5.03)
Midline pain only (H) Schwarzer 1995 [34]	0.03 (0.00–0.14)	---	---	---
Facet joint				
Revel’s suggested rule: 5 of 7 positive findings Manchikanti 2000 [35]	0.13 (0.07–0.22)	0.84 (0.76–0.90)	0.8 (0.4–1.7)	1.03 (0.92–1.16)
Age more than 65 years (H)	0.22 (0.14–0.32)	0.85 (0.77–0.91)	1.5 (0.8–2.6)	0.92 (0.80–1.05)
Pain relieved in recumbent position (P)	0.94 (0.86–0.98)	0.17 (0.10–0.25)	1.1 (1.0–1.2)	0.39 (0.18–0.96)
Pain not increased with cough (P)	0.90 (0.82–0.95)	0.13 (0.08–0.21)	1.0 (0.9–1.1)	0.76 (0.34–1.66)
Pain not increased with forward flexion (P)	0.16 (0.09–0.25)	0.82 (0.73–0.88)	0.9 (0.5–1.6)	1.03 (0.91–1.17)
Pain not increased with rising from flexion (P)	0.55 (0.44–0.65)	0.48 (0.39–0.58)	1.1 (0.8–1.4)	0.94 (0.70–1.26)
Pain not increased with hyperextension (P)	0.10 (0.05–0.18)	0.86 (0.78–0.92)	0.7 (0.3–1.2)	1.05 (0.95–1.16)
Pain not increased with extension/rotation (P)	0.68 (0.57–0.77)	0.30 (0.22–0.40)	1.0 (0.8–1.2)	1.07 (0.71–1.61)
Studies supporting items of Revel’s suggested rule				
Pain relieved in recumbent position (P) Single block Revel 1998 [38]	0.96 (0.71–1.00)	0.48 (0.30–0.67)	1.9 (1.3–2.7)	0.07 (0.01–1.15)
Pain relieved in recumbent position (P) Single block Revel 1992 [37]	0.63 (0.41–0.82)	0.76 (0.52–0.92)	2.7 (1.1–6.3)	0.48 (0.27–0.87)
Studies not supporting items of Revel’s suggested rule				
Age more than 65 years (H) Manchikanti 1999 [36]	0.19 (0.10–0.32)	0.66 (0.54–0.78)	0.6 (0.3–1.1)	1.21 (0.98–1.51)
Age more than 61 years (H) Manchikanti 2008 [41]	0.19 (0.12–0.29)	0.75 (0.69–0.81)	0.8 (0.5–1.3)	1.07 (0.94–1.12)
No pain with extension/rotation (P) Schwarzer 1994 [42]	0.0 (0.0–0.13)	0.88 (0.82–0.93)	0.0 (---)	1.13 (1.07–1.20)
No pain with hyperextension (P) Fairbank 1981 [43]	0.36 (0.16–0.61)	0.36 (0.15–0.65)	0.6 (0.2–1.3)	1.77 (0.74–4.24)
Revel’s suggested rule Single block^a^ Laslett 2004 [40]	0.11 (0.39–2.8)	0.91 (0.83–0.95)	1.2 (0.4–4.3)	0.98 0.84–1.13
Additional findings reported by more than one study				
Traumatic onset (H) Manchikanti 2000 [35]	0.48 (0.37–0.59)	0.50 (0.41–0.59)	1.0 (0.7–1.3)	1.05 (0.80–1.37)
Traumatic onset (H) Manchikanti 1999 [36]	0.54 (0.40–0.67)	0.47 (0.35–0.60)	1.0 (0.7–1.4)	0.99 (0.67–1.44)
No centralization (P) Single block^a^ Young 2003 [32]	1.00 (0.78–1.0)	0.11 (0.02–0.44)	1.3 (0.9–1.4)	NA
No centralization (P) Single block^a^ Laslett 2006 [39]	1.00 (0.74–1.0)	0.17 (0.11–0.27)	1.2 (1.1–1.3)	NA
Sacroiliac joint				
Laslett composite: no centralization and 3 of 5 positive findings: distraction, compression, thigh thrust, Gaenslen’s test, sacral thrust (P)^a^ Laslett 2003 [44]	0.91 (0.62–0.98)	0.87 (0.68–0.95)	7.0 (2.4–20.4)	0.11 (0.02–0.68)
Laslett rule: 3 of 5 positive findings alone (P)^a^ Laslett 2003 [44]	0.91 (0.62–0.98)	0.78 (0.61–0.89)	4.2 (2.1–8.2)	0.12 (0.02–0.76)
Studies supporting items of Laslett’s rule				
van der Wurff composite 3 out of 5 positive findings: distraction, compression, thigh thrust, Gaenslen’s test, Patrick’s test (P) van der Wurff 2006 [45]	0.85 (0.72–0.99)	0.79 (0.65–0.93)	4.0 (2.0–7.9)	0.19 (0.07–0.47)
Stanford composite 3 out of 5 positive findings: Patrick’s test, thigh thrust, Gaenslen’s test, compression, sacral thrust (P) Stanford 2010 [47]	0.82 (0.52–0.95)	0.57 (0.37–0.74)	1.9 (1.1–3.2)	0.32 (0.09–1.19)
Ozgocmen composite 3 out of 5 positive findings: Patrick’s test, thigh thrust, Gaenslen’s test, Mennell, sacral thrust (P) Ozgocmen 2008 [48]	0.45 (0.18–0.75)	0.89 (0.71–0.97)	4.4 (1.3–15.4)	0.62 (---)
No centralization (P) Single block^a^ Young 2003 [32]	0.92 (0.76–0.98)	0.23 (0.12–0.41)	1.2 (1.0–1.5)	0.33 (0.08–1.45)
Studies not supporting items of Laslett’s rule				
Gaenslen’s test (P) Single block Dreyfuss 1996 [46]	0.67 (0.52–0.79)	0.35 (0.22–0.50)	1.0 (0.8–1.4)	0.95 (0.53–1.72)
Thigh thrust (P) Single block Dreyfuss 1996 [46]	0.42 (0.29–0.57)	0.45 (0.31–0.60)	0.8 (0.5–1.2)	1.28 (0.84–1.96)
Sacral thrust (P) Single block Dreyfuss 1996 [46]	0.51 (0.36–0.66)	0.40 (0.25–0.57)	0.9 (0.6–1.2)	1.22 (0.76–1.96)
Additional findings reported by more than one study				
Dominant pain in SIJ without tuber area (H) Van der Wurff 2006 [49]	0.89 (0.72–0.96)	0.79 (0.62–0.89)	4.2 (2.1–8.20)	0.14 (0.05–0.42)
PSIS pointing Single block Dreyfuss 1996 [46]	0.71 (0.57–0.82)	0.48 (0.33–0.63)	1.4 (1.0–1.9)	0.61 (0.35–1.07)
Disc herniation with nerve root involvement				
Hancock rule L4 nerve, 3 out of 4 positive findings: corresponding dermatomal pain location, sensory deficits, reflex and motor weakness Hancock 2011 [52]	0.50 (0.21–0.79)	0.90 (0.85–0.93)	5.0 (?)	0.01 (?)
Hancock rule L5 nerve, 3 out of 4 positive findings: Corresponding dermatomal pain location, sensory deficits, reflex and motor weakness Hancock 2011 [52]	0.37 (0.28–0.46)	0.83 (0.76–0.88)	2.2 (?)	0.76 (?)
Hancock rule S1 nerve, 3 out of 4 positive findings: corresponding dermatomal pain location, sensory deficits, reflex and motor weakness Hancock 2011 [52]	0.28 (0.21–0.35)	0.94 (0.88–0.98)	4.7 (?)	0.77 (?)
L4 dermatomal pain location only (P) L3 disc	0.39 (0.14–0.68)	0.97 (0.94–0.99)	13.0 (?)	0.63 (?)
L5 pain dermatomal location only (P) L4 disc	0.25 (0.18–0.34)	0.92 (0.86–0.96)	3.2 (?)	0.79 (?)
S1 pain dermatomal location only (P) L5 disc	0.22 (0.16–0.29)	0.98 (0.94–1.00)	11.0 (?)	0.80 (?)
L4 location sensory loss (P) L3 disc	0.42 (0.15–0.72)	0.74 (0.69–0.79)	1.6 (0.8–3.3)	0.79 (0.49–1.28)
L5 location sensory loss (P) L4 disc	0.60 (0.51–0.69)	0.54 (0.45–0.62)	1.3 (1.0–1.6)	0.75 (0.58–0.97)
S1 location sensory loss (P) L5 disc	0.59 (0.51–0.66)	0.60 (0.50–0.69)	1.5 (1.1–1.9)	0.69 (0.54–0.87)
Patellar reflex weakness (P) L3 disc	0.50 (0.21–0.79)	0. 83 (0.78–0.87)	2.9 (0.6–5.5)	0.60 (0.34–1.06)
Achilles reflex weakness (P) L5 disc	0.48 (0.40–0.56)	0.83 (0.75–0.90)	2.9 (1.8–4.5)	0.62 (0.52–0.74)
Quadriceps weakness (P) L3 disc	0.67 (0.35–0.90)	0.40 (0.34–0.46)	1.1 (0.7–1.7)	0.84 (0.37–1.89)
Tibialis anterior weakness (P) L4 disc	0.46 (0.37–0.55)	0.70 (0.63–0.77)	1.6 (1.1–2.1)	0.77 (0.63–0.93)
Peroneal weakness (P) L4 disc	0.50 (0.41–0.59)	0.68 (0.60–0.75)	1.6 (1.2–2.1)	0.73 (0.60–0.90)
Ext. hallucis longus weakness (P) L4 disc	0.54 (0.44–0.63)	0.64 (0.56–0.72)	1.5 (1.2–2.0)	0.72 (0.58–0.90)
Calf weakness (P) L5 disc	0.30 (0.23–0.38)	0.63 (0.53–0.72)	0.8 (0.6–1.1)	1.11 (0.94–1.33)
Studies supporting items of the Hancock rule				
All 3 findings positive: sensory loss, paresis, loss of reflexes (P) any nerve Vroomen 1998 [74]	0.31 (0.14–0.56)	0.93 (0.83–0.97)	4.3 (1.3–14.1)	0.74 (0.53–1.04)
Dermatomal pain location (H) any nerve Vroomen 2002 [53]	0.89 (0.84–0.93)	0.31 (0.24–0.40)	1.3 (1.1–1.5)	0.34 (0.20–0.58)
Pain location (H) corresponding S1 nerve Bertilson 2010 [54]	0.55 (0.28–0.79)	0.76 (0.63–0.86)	2.3 (1.1–4.7)	0.60 (0.31–1.16)
L5 location sensory loss (P) disc L4 Kerr 1988 [55]	0.30 (0.19–0.44)	0.86 (0.71–0.94)	2.2 (0.9–5.4)	0.81 (0.65–1.02)
S1 location sensory loss (P) disc L5 Kerr 1988 [55]	0.45 (0.31–0.59)	0.86 (0.71–0.94)	3.2 (1.3–7.7)	0.64 (0.48–0.86)
Anterior thigh sensory loss (P) L2-L4 nerves Suri 2010 [56]	0.08 (1.01–0.27)	0.96 (0.82–1.00)	2.3 (0.23–24.2)	0.95 (0.83–1.09)
Anterior thigh sensory loss (P) L2 nerve Suri 2010 [56]	0.50 (0.01–0.99)	0.96 (0.86–1.00)	12.5 (1.8–87.0)	0.52 (0.13–2.08)
Medial knee sensory loss (P) L2-L4 nerves Suri 2010 [56]	0.17 (0.05–0.37)	0.96 (0.82–1.00)	4.7 (0.6–39.00)	0.86 (0.71–1.05)
Medial ankle sensory loss (P) L2-L4 nerves Suri 2010 [56]	0.17 (0.05–0.37)	1.00 (0.88–1.00)	NA	0.83 (0.69–1.01)
Medial ankle sensory loss (P) L4 nerve Suri 2010 [56]	0.31 (0.09–0.61)	1.00 (0.91–1.00)	NA	0.69 (0.48–0.99)
Medial foot sensory loss (P) L4 disc Gurdjian 1961 [57]	0.13 (0.11–0.16)	0.94 (0.92–0.96)	2.3 (1.5–3.3)	0.92 (0.89–0.96)
Lateral foot sensory loss (P) L5-S1 nerves Suri 2010 [56]	0.21 (0.08–0.41)	0.92 (0.73–0.99)	2.6 (0.6–11.6)	0.86 (0.68–1.08)
Lateral foot sensory loss(P) L5 disc Gurdjian 1961 [57]	0.23 (0.20–0.27)	0.90 (0.87–0.92)	2.3 (1.7–3.1)	0.85 (0.81–0.90)
S1 location sensory loss (P) L5 disc Kerr 1988 [49]	0.47 (0.33–0.61)	0.86 (0.71–0.94)	3.3 (1.4–8.0)	0.61 (0.46–0.83)
Patellar reflex weakness (P) L4 nerve Suri 2010 [56]	0.39 (0.18–0.65)	0.95 (0.84–0.99)	7.7 (1.7–35.0)	0.65 (0.42–1.00)
Patellar reflex weakness (P) L3 disc Knutsson 1961 [58]	1.00 (0.34–1.00)	0.84 (0.78–0.89)	6.4 (4.6–9.0)	NA
Achilles reflex weakness (P) L5-S1 nerves Suri 2010 [56]	0.29 (0.13–0.49)	0.96 (0.80–1.00)	7.1 (1.0–53.2)	0.74 (0.58–0.95)
Achilles reflex weakness (P) L5 nerve Suri 2010 [56]	0.33 (0.16–0.56)	0.91 (0.78–0.97)	3.9 (1.1–13.8)	0.73 (0.52–1.03)
Achilles reflex weakness (P) L5 disc Gurdjian 1961 [57]	0.56 (0.52–0.60)	0.75 (0.71–0.79)	2.3 (1.9–2.7)	0.58 (0.52–0.64)
Achilles reflex weakness (P) L5 disc Kerr 1988 [55]	0.87 (0.75–0.94)	0.89 (0.75–0.96)	7.9 (3.1–19.9)	0.14 (0.07–0.31)
Achilles reflex weakness (P) L5 disc Knutsson 1961 [58]	0.78 (0.67–0.86)	0.65 (0.55–0.73)	2.2 (1.7–2.9)	0.34 (0.22–0.54)
Reflex absence ankle/knee (P) any nerve Vroomen 2002 [53]	0.14 (0.09–0.21)	0.93 (0.88–0.97)	2.2 (1.0–4.8)	0.91 (0.84–0.99)
Sit to stand weakness (P) L3 nerve Suri 2010 [56]	0.50 (0.19–0.81)	0.77 (0.62–0.89)	2.2 (1.00–5.0)	0.65 (0.34–1.23)
Sit to stand weakness (P) L4 nerve Suri 2010 [56]	0.54 (0.25–0.81)	0.80 (0.65–0.91)	2.8 (1.2–6.1)	0.57 (0.31–1.05)
Heel raise weakness (P) L5-S1 nerves Suri 2010 [56]	0.14 (0.04–0.32)	0.96 (0.80–1.00)	3.5 (0.4–28.9)	0.90 (0.76–1.06)
Great toe ext. weakness (P) L5 nerve Suri 2010 [56]	0.61 (0.36–0.83)	0.86 (0.71–0.95)	4.4 (1.8–10.8)	0.45 (0.25–0.82)
Great toe ext. weakness (P) L4 disc Knutsson 1961 [58]	0.75 (0.65–0.83)	0.53 (0.43–0.63)	1.6 (1.3–2.1)	0.47 (0.31–0.71)
Ankle dorsiflexion weakness (P) L4 disc Kerr 1988 [55]	0.60 (0.46–0.72)	0.89 (0.75–0.96)	5.4 (2.1–13.9)	0.45 (0.31–0.64)
Ankle plantarflexion weakness (P) L5 disc Kerr 1988 [55]	0.13 (0.06–0.25)	1.00 (0.90–1.00)	NA	0.87 (0.78–0.97)
Paresis not specified (P) any nerve Vroomen 2002 [53]	0.27 (0.21–0.35)	0.93 (0.88–0.97)	4.1 (2.0–8.4)	0.78 (0.70–0.87)
Studies not supporting items of the Hancock rule				
Pain location (H) corresponding L4 nerve Bertilson 2010 [54]	0.00 (0.00–0.32)	0.85 (0.73–0.92)	0.0 (NA)	1.18 (1.05–1.32)
Pain location (H) corresponding L5 nerve Bertilson 2010 [54]	0.78 (0.55–0.91)	0.28 (0.17–0.43)	1.1 (0.79–1.47)	0.80 (0.30–2.14)
Non-specific sensory deficits (P) any disc level Stankovic 1999 [59]	0.56 (0.42–0.68)	0.40 (0.28–0.53)	0.9 (0.7–1.3)	1.12 (0.71–1.75)
Non-specific sensory deficits (P) any disc level Vucetic 1996 [60]	0.45 (0.37–0.53)	0.69 (0.39–0.91)	1.5 (0.6–3.3)	0.80 (0.54–1.18)
Sensory loss not specified (P) L3-L5 discs Kosteljanetz 1984 [63]	0.60 (0.47–0.73)	0.57 (0.41–0.72)	1.4 (0.9–2.1)	0.69 (0.46–1.05)
Sensory loss not specified (P) L3-L5 disc Knutsson 1961 [58]	0.28 (0.22–0.36)	0.65 (0.41–0.85)	0.8 (0.4–1.6)	1.10 (0.79–1.54)
Sensory loss not specified (P) L5 or S1 nerve Albeck 1996 [61]	0.67 (0.54–0.79)	0.42 (0.20–0.67)	1.2 (0.8–1.8)	0.78 (0.41–1.47)
Great toe sensory loss (P) L5-S1 nerves Suri 2010 [56]	0.18 (0.06–0.37)	0.87 (0.66–0.97)	1.4 (0.4–5.1)	0.94 (0.75–1.19)
Hypesthesia (P) any nerve Vroomen 2002 [53]	0.28 (0.21–0.36)	0.66 (0.56–0.74)	0.8 (0.6–1.2)	1.09 (0.93–1.29)
Hypalgesia (P) any nerve Vroomen 2002 [53]	0.17 (0.11–0.24)	0.84 (0.77–0.90)	1.1 (0.6–1.9)	0.98 (0.88–1.09)
Hypesthesia (P) L5 or S1 nerve (P) Albeck 1996 [61]	0.67 (0.54–0.79)	0.42 (0.20–0.67)	1.2 (0.8–1.8)	0.78 (0.41–1.47)
Disturbed touch sensibility (P) corresponding L4 nerve Bertilson 2010 [54]	0.13 (0.02–0.47)	0.75 (0.62–0.85)	0.5 (0.1–3.4)	1.16 (0.86–1.57)
Disturbed touch (P) corresponding L5 nerve Bertilson 2010 [54]	0.22 (0.09–0.45)	0.51 (0.37–0.65)	0.5 (0.2–1.1)	1.52 (1.04–2.23)
Disturbed touch sensibility (P) corresponding S1 nerve Bertilson 2010 [54]	0.36 (0.15–0.65)	0.68 (0.54–0.79)	1.1 (0.5–2.7)	0.94 (0.58–1.52)
Disturbed pain sensibility (P) corresponding L4nerve Bertilson 2010 [54]	0.00 (0.00–0.32)	0.45 (034.–0.60)	0.0 (NA)	2.1 (1.59.–2.82)
Disturbed pain sensibility (P) corresponding L5 nerve Bertilson 2010 [54]	0.44 (0.25–0.66)	0.40 (0.26–0.54)	0.7 (0.4–1.3)	1.4 (0.81.–2.45)
Disturbed pain sensibility (P) corresponding S1 nerve Bertilson 2010 [54]	0.36 (0.15–0.65)	0.58 (0.44–0.71)	0.9 (0.4–2.0)	1.10 (0.66.–1.81)
Patellar reflex weakness (P) L3 disc Gurdjian 1961 [57]	0.02 (0.00–0.12)	0.92 (0.91–0.94)	0.3 (0.0–2.2)	1.06 (1.01–1.11)
Achilles reflex weakness (P) L3-L5 disc Spangfort 1972 [62]	0.31 (0.29–0.33)	0.80 (0.76–0.84)	1.6 (1.3–2.0)	0.86 (0.81–0.91)
Reflex weakness not specified (P) L5 or S1 nerve Albeck 1996 [61]	0.61 (0.47–0.73)	0.63 (0.38–0.84)	1.7 (0.9–3.1)	0.62 (0.39–0.99)
Disturbed reflexes not specified (P) corresponding L4 nerve Bertilson 2010 [54]	0.38 (0.14–0.69)	0.64 (0.51–0.76)	1.0 (0.4–2.7)	1.00 (0.55–1.73)
Disturbed reflexes not specified (P) corresponding L5 nerve Bertilson 2010 [54]	0.61 (0.39–0.80)	0.56 (0.41–0.70)	1.4 (0.8–2.3)	0.70 (0.37–1.32)
Disturbed reflexes not specified (P) corresponding S1 nerve Bertilson 2010 [54]	0.27 (0.10–0.57)	0.48 (0.35–0.62)	0.5 (0.2–1.4)	1.52 (0.95–2.41)
Reflex weakness not specified (P) any disc level Stankovic 1999 [59]	0.46 (0.33–0.60)	0.70 (0.56–0.80)	1.5 (0.9–2.5)	0.77 (0.57–1.05)
Reflex weakness not specified (P) any level Vucetic 1996 [60]	0.35 (0.28–0.44)	0.77 (0.46–0.95)	1.5 (0.6–4.2)	0.84 (0.61–1.16)
Heel walk weakness (P) L5-S1 nerves Suri 2010 [56]	0.14 (0.04–0.32)	0.80 (0.59–0.93)	0.7 (0.2–2.3)	1.08 (0.84–1.38)
Great toe ext. weakness (P) L5-S1 nerves Suri 2010 [56]	0.38 (0.21–0.58)	0.80 (0.59–0.93)	1.9 (0.8–4.7)	0.78 (0.55–1.10)
Ankle dorsiflexion weakness (P) any level Vucetic 1996 [60]	0.29 (0.22–0.37)	0.77 (0.46–0.95)	1.2 (0.4–3.5)	0.93 (0.68–1.27)
Foot drop (P) L4 disc Gurdjian 1961 [51]	0.01 (0.00–0.01)	0.93 (0.86–0.97)	0.1 (0.0–0.3)	1.07 (1.01–1.13)
Extensor hallucis longus weakness (P) L4 disc Gurdjian 1961 [57]	0.20 (0.17–0.23)	0.88 (0.86–0.91)	1.8 (1.3–2.3)	0.90 (0.86–0.95)
Ankle dorsiflex weakness (P) L3-L5 disc Spangfort 1972 [62]	0.30 (0.28–0.32)	0.66 (0.61–0.71)	0.9 (0.8–1.1)	1.06 (0.97–1.15)
Disturbed motor function not specified (P) corresponding L4 nerve Bertilson 2010 [54]	0.50 (0.22–0.78)	0.53 (0.40–0.66)	1.1 (0.5–2.2)	0.95 (0.45–1.98)
Disturbed motor function not specified (P) corresponding L5 nerve Bertilson 2010 [54]	0.56 (0.34–0.75)	0.42 (0.28–0.57)	1.0 (0.6–1.6)	1.06 (0.57–1.98)
Disturbed motor function not specified (P) corresponding S1nerve Bertilson 2010 [54]	0.36 (0.15–0.65)	0.62 (0.48–0.74)	1.0 (0.4–2.3)	1.03 (0.63–1.69)
Motor weakness not specified (P) L5 or S1 nerve Albeck 1996 [61]	0.34 (0.23–0.48)	0.47 (0.24–0.71)	0.7 (0.4–1.1)	1.38 (0.83–2.30)
Motor weakness not specified (P) L3-L5 disc Knutsson 1961 [58]	0.62 (0.54–0.69)	0.50 (0.27–0.73)	1.2 (0.8–2.0)	0.77 (0.70–1.49)
Motor weakness not specified (P) L3-L5 discs Kosteljanetz 1984 [63]	0.47 (0.33–0.60)	0.52 (0.36–0.68)	1.0 (0.6–1.5)	1.02 (0.46–1.05)
Motor weakness not specified (P) any disc level Stankovic 1999 [59]	0.60 (0.46–0.72)	0.38 (0.26–0.51)	1.0 (0.7–1.3)	1.07 (0.66–1.7)
Additional findings reported by more than one study				
Straight Leg Raise (P) L5 or S1 nerves Albeck 1996 [61]	0.83 (0.72–0.91)	0.21 (0.09–0.43)	1.1 (0.8–1.4)	0.78 (0.28–2.20)
Straight Leg Raise (P) L5 or S1 nerves Suri 2010 [56]	0.69 (0.51–0.83)	0.84 (0.65–0.94)	4.3 (1.7–11.0)	0.37 (0.21–0.65)
Straight Leg Raise (P) L5 nerve Suri 2010 [56]	0.67 (0.44–0.84)	0.67 (0.50–0.80)	2.0 (1.1–3.5)	0.50 (0.25–1.0)
Straight Leg Raise (P) L3-L5 discs Knutsson 1961 [58]	0.96 (0.91–0.98)	0.10 (0.03–0.30)	1.1 (0.9–1.2)	0.43 (0.10–1.94)
Straight Leg Raise (P) L3-L5 discs Spangfort 1972 [62]	0.97 (0.96–0.97)	0.11 (0.08–0.15)	1.1 (1.1–1.1)	0.29 (0.20–0.42)
Straight Leg Raise (P) L4 or L5 discs Kerr 1988 [55]	0.98 (0.93–0.99)	0.44 (0.30–0.60)	1.8 (1.3–2.4)	0.05 (0.01–0.19)
Straight Leg Raise (P) L4 or L5 discs Gurdjian 1961 [57]	0.82 (0.80–0.84)	0.45 (0.35–0.56)	1.5 (1.2–1.8)	0.40 (0.30–0.52)
Straight Leg Raise (P) L3-L5 discs Gurdjian 1961 [57]	0.81 (0.78–0.83)	0.37 (0.23–0.54)	1.2 (1.0–1.7)	0.52 (0.34–0.81)
Straight Leg Raise (P) L4-L5 discs Demircan 2002 [71]	0.97 (0.94–0.99)	0.82 (0.73–0.89)	5.4 (3.6–8.2)	0.03 (0.01–0.08)
Straight Leg Raise (P) L3-L5 discs Kosteljanetz 1984 [63]	0.79 (0.66–0.88)	0.48 (0.32–0.63)	1.5 (1.1–2.1)	0.45 (0.25–0.81)
Straight Leg Raise (P) any disc level Charnley 1951 [72]	0.78 (0.68–0.86)	0.64 (0.39–0.84)	2.2 (1.1–4.5)	0.34 (0.19–0.60)
Straight Leg Raise(P) any disc level Kosteljanetz 1988 [70]	0.89 (0.76–0.96)	0.14 (0.00–0.58)	1.0 (0.8–1.4)	0.78 (0.11–5.71)
Straight Leg Raise (P) any disc level Hakelius 1972 [64]	0.96 (0.95–0.97)	0.14 (0.11–0.18)	1.1 (1.1–1.2)	0.27 (0.19–0.38)
Straight leg raise (P) any nerve Vroomen 2002 [53]	0.64 (0.56–0.71)	0.57 (0.48–0.65)	1.5 (1.2–1.9)	0.64 (0.49–0.83)
Straight leg raise (P) any disc level Majlesi 2008 [73]	0.53 (0.37–0.67)	0.89 (0.75–0.96)	4.9 (1.8–12.9)	0.53 (0.37–0.76)
Straight leg raise (P) any disc level Haldeman 1988 [68]	0.44 (0.33–0.57)	0.78 (0.61–0.89)	2.0 (1.0–4.2)	0.71 (0.53–0.94)
Straight leg raise (P) any disc level^a^ Meylemans 1988 [67]	0.35 (0.26–0.44)	1.00 (0.92–1.00)	NA	0.65 (0.57–0.75)
Crossed SLR (P) L5 or S1 nerves Suri 2010 [56]	0.07 (0.02–0.22)	0.96 (0.81–0.99)	1.7 (0.2–18.0)	0.97 (0.85–1.1)
Crossed SLR (P) L4 or L5 disc Kerr 1988 [55]	0.43 (0.34–0.53)	0.97 (0.86–0.99)	15.6 (2.2–109.1)	0.58 (0.49–0.74)
Crossed SLR (P) L3-L5 discs Spangfort 1972 [62]	0.23 (0.21–0.25)	0.88 (0.84–0.91)	2.0 (1.5–2.6)	0.87 (0.83–0.91)
Crossed SLR (P) L3-L5 discs Knutsson 1961 [58]	0.25 (0.18–0.32)	0.95 (0.74–1.00)	4.7 (0.7–32.2)	0.80 (0.69–0.91)
Crossed SLR (P) L4-L5 discs Poiraudeau 2001 [69]	0.29 (0.16–0.45)	0.83 (0.66–0.93)	1.7 (0.7–4.0)	0.86 (0.68–1.10)
Crossed SLR (P) any disc level Kosteljanetz 1988 [70]	0.24 (0.13–0.40)	1.00 (0.59–1.00)	NA	0.76 (0.64–0.89)
Crossed SLR (P) any disc level Stankovic 1999 [59]	0.29 (0.18–0.42)	0.87 (0.75–0.93)	2.2 (1.0–4.9)	0.82 (0.67–1.00)
Slump (P) any disc level Majlesi 2008 [73]	0.84 (0.74–0.90)	0.83 (0.73–0.90)	4.9 (?)	0.19 (?)
Slump (P) any disc level Stankovic 1999 [59]	0.94 (0.84–0.98)	0.23 (0.13–0.36)	1.2 (1.0–1.4)	0. 26 (0.08–0.85)
Spinal Stenosis				
Cook rule: 3 of 5 positive findings Cook 2011 [76]	0.29 (0.27–0.31)	0.88 (0.87–0.90)	2.5 (2.0–3.1)	0.80 (0.76–0.85)
Age more than 48 years (H)	0.88 (0.85–0.89)	0.49 (0.47–0.50)	1.7 (1.6–1.8)	0.25 (0.21–0.32)
Bilateral symptoms (H)	0.03 (0.02–0.04)	0.98 (0.98–0.99)	2.3 (1.1–4.8)	0.98 (0.97–0.99)
Leg pain worse than back pain (H)	0.16 (0.14–0.18)	0.92 (0.91–0.93)	2.1 (1.5–2.8)	0.91 (0.87–0.94)
Pain with walking/standing (H)	0.67 (0.64–0.69)	0.44 (0.42–0.46)	1.2 (1.1–1.3)	0.75 (0.66–0.86)
Sitting relieves pain (H)	0.26 (0.24–0.29)	0.86 (0.84–0.88)	1.9 (1.5–2.3)	0.86 (0.82–0.91)
Studies supporting items of the Cook rule				
Age more than 50 years (H) Konno 2007 [84]	0.95 (0.90–0.98)	0.79 (0.70–0.86)	4.6 (3.10–6.81)	0.06 (0.03–0.13)
Bilateral pain (H) Ljunggren 1991 [78]	0.51 (0.40–0.62)	0.92 (0.85–0.96)	6.3 (3.15–12.74)	0.54 (0.43–0.68)
Severe leg pain (H) Katz 1995 [79]	0.65 (0.51–0.79)	0.67 (0.51–0.83)	2.0 (?)	0.52 (?)
Symptoms extending down the legs when walking (P) Jensen 1989 [80]	0.63 (0.31–0.86)	0.80 (0.55–0.93)	3.1 (0.99–9.82)	0.47 (0.19–1.19)
Leg pain or numbness (H) Konno 2007 [84]	0.94 (0.90–0.98)	0.12 (0.07–0.20)	1.1 (0.99–1.18)	0.41 (0.17–1.01)
Radiating leg pain (disc disease with spinal stenosis) (H) Roach 1997 [81]	0.94 (?)	0.21 (?)	1.2 (?)	0.29 (?)
Symptoms worse by standing (H) Konno 2007 [84]	0.85 (0.78–0.90)	0.75 (0.66–0.83)	3.4 (2.40–4.88)	0.20 (0.14–0.31)
Symptoms exacerbated when standing up (H) Sugioka 2008 [82]	0.92 (0.87–0.95)	0.21 (0.15–0.27)	1.2 (1.06–1.27)	0.39 (0.22–0.69)
Walking or standing worst posture (H) Fritz 1997 [83]	0.88 (0.71–0.96)	0.33 (0.16–0.56)	1.3 (0.93–1.89)	0.35 (0.10–1.21)
Symptoms worse walking and relieved by rest (H) Konno 2007 [84]	0.94 (0.89–0.97)	0.81 (0.73–0.88)	5.1 (3.34–7.71)	0.07 (0.04–0.14)
Pseudoclaudication (H) Roach 1997 [81]	0.63 (?)	0.71 (?)	2.2 (?)	0.52 (?)
Sitting best posture (H) Fritz 1997 [83]	0.89 (0.71–0.96)	0.39 (0.20–0.61)	1.5 (0.98–2.15)	0.29 (0.09–1.0)
Symptoms improve when seated (H) Katz 1995 [79]	0.53 (0.37–0.67)	0.83 (0.70–0.96)	3.1 (?)	0.58 (?)
Studies not supporting items of the Cook rule				
Pain below buttocks (H) Katz 1995 [79]	0.88 (0.78–0.98)	0.34 (0.18–0.50)	1.3 (?)	0.64 (?)
Leg pain with walking that is relieved by sitting (H) Fritz 1997 [83]	0.81 (0.62–0.91)	0.16 (0.55–0.38)	0.96 (0.73–1.26)	1.2 (0.33–4.49)
Pain worse when walking (H) Katz 1995 [79]	0.71 (0.57–0.85)	0.30 (0.14–0.46)	1.0 (?)	0.97 (?)
Intermittent claudication (H) Sugioka 2008 [82]	0.73 (0.66–0.79)	0.38 (0.31–0.46)	1.2 (1.02–1.38)	0.70 (0.52–0.95)
Pain occurs while walking (H) Sugioka 2008 [82]	0.83 (0.77–0.87)	0.27 (0.21–0.34)	1.1 (1.01–1.26)	0.64 (0.44–0.97)
Additional findings reported by more than one study				
Symptoms improved by bending forward (H) Konno 2007 [84]	0.72 (0.64–0.79)	0.92 (0.85–0.96)	8.8 (4.48–17.15)	0.30 (0.23–0.40)
Symptoms improved by bending forward (H) Sugioka 2008 [82]	0.43 (0.36–0.50)	0.75 (0.69–0.81)	1.7 (1.28–2.36)	0.76 (0.66–0.88)
No pain with flexion (P) Katz 1995 [79]	0.79 (0.67–0.91)	0.44 (0.27–0.61)	1.4 (?)	0.48 (?)
Walking easier bending forward (H) Sugioka 2008 [82]	0.55 (0.48–0.62)	0.61 (0.53–0.68)	1.4 (1.12–1.75)	0.74 (0.61–0.90)
Improved treadmill walking tolerance bending forward (Distinguish from PVD) (P) Dong 1989 [85]	0.58 (0.36–0.77)	0.82 (0.52–0.95)	3.2 (0.85–11.81)	0.52 (0.28–0.93)
Earlier onset of symptoms with level treadmill walking vs inclined (P) Fritz 1997 [83]	0.65 (0.46–0.81)	0.84 (0.62–0.94)	4.1 (1.41–12.14)	0.41 (0.23–0.72)
Symptoms improved by walking uphill (Distinguish from PVD) (H) Dong 1989 [85]	0.16 (0.55–0.38)	1.0 (0.74–1.0)	NA	0.84 (0.69.–1.02)
Thigh pain with extension (P) Katz 1995 [79]	0.51 (0.36–0.66)	0.69 (0.53–0.85)	1.6 (?)	0.71 (?)
Symptoms induced by bending backward (H) Konno 2007 [84]	0.62 (0.54–0.70)	0.48 (0.39–0.58)	1.2 (0.95–1.52)	0.78 (0.58–1.05)
Gait abnormality (ataxic, wide based, poor coordination) (P) Cook 2011 [76]	0.29 (0.27–0.32)	0.81 (0.79–0.83)	1.6 (1.2–1.9)	0.87 (0.82–0.92)
Wide based gait (P) Katz 1995 [79]	0.43 (0.28–0.58)	0.97 (0.91–1.0)	14.3 (?)	0.59 (?)
Spondylolisthesis				
Studies supporting a diagnostic rule				
Manual hypermobility test positive (P) Fritz 2005 [87]	0.46 (0.30–0.64)	0.81 (0.60–0.92)	2.4 (0.9–6.4)	0.66 (0.44–0.99)
Lack of manual hypomobility test positive (P) Fritz 2005 [87]	0.43 (0.27–0.61)	0.95 (0.77–0.99)	9.0 (1.3–63.9)	0.60 (0.43–0.84)
Lack of manual hypomobility test positive and flexion ROM > 53° (P) Fritz 2005 [87]	0.29 (0.13–0.46)	0.98 (0.91–1.00)	12.8 (0.8–211.6)	0.72 (0.55–0.94)
Manual flexion hypermobility test positive (P) rotation Abbott 2005 [88]	0.05 (0.01–0.36)	0.99 (0.96–1.00)	4.1 (0.2–80.3)	0.96 (0.83–1.11)
Manual flexion hypermobility test positive (P)translation Abbott 2005 [88]	0.05 (0.01–0.22)	0.99 (0.97–1.00)	8.7 (0.6–134.7)	0.96 (0.88–1.05)
Manual extension hypermobility test positive (P) rotation Abbott 2005 [88]	0.22 (0.06–0.55)	0.97 (0.94–0.99)	8.4 (1.9–37.6)	0.80 (0.56–1.13)
Manual extension hypermobility test positive (P) translation Abbott 2005 [88]	0.16 (0.06–0.38)	0.98 (0.94–0.99)	7.1 (1.7–29.2)	0.86 (0.71–1.05)
Slipping by palpation (P) Kalpakcioglu 2009 [90]	0.88 (0.80–0.93)	1.00 (0.89–1.00)	NA	0.12 (0.07–0.20)
Slipping by palpation (P) Collaer 2006 [91]	0.60 (0.15–0.95)	0.87 (0.73–0.96)	4.7 (1.6–13.9)	0.46 (0.16–1.35)
Passive lumbar extension test (P) Kasai 2006 [89]	0.84 (0.70–0.93)	0.90 (0.82–0.95)	8.8 (4.5–17.3)	0.18 (0.08–0.37)
Passive lumbar extension test (P) Ferrari 2014 [92]	0.44 (0.29–0.59)	0.86 (0.67–0.95)	3.2 (1.1–9.7)	0.65 (0.47–0.90)
Studies not supporting a diagnostic rule				
None				
Additional findings reported by more than one study				
Slipping by inspection (P) Kalpakcioglu 2009 [90]	0.21 (0.14–0.30)	1.00 (0.89–1.00)	NA	0.79 (0.71–0.87)
Slipping and Sill sign by inspection and palpation (P) Ahn 2015 [93]	0.81 (0.65–0.91)	0.89 (0.79–0.95)	7.4 (3.6–15.2)	0.21 (0.10–0.44)
Aberrant movements (P) Fritz 2005 [87]	0.18 (0.08–0.36)	0.90 (0.71–0.97)	1.9 (0.4–8.7)	0.91 (0.73–1.13)
Aberrant movements (P) Sundell 2013 [94]	0.69 (0.42–0.87)	0.50 (0.25–0.74)	1.4 (0.7–2.7)	0.62 (0.23–1.66)
Fracture				
The Henschke rule 1 out of 3 positive findings: age >70 years, significant trauma, prolonged use of corticosteroids (H) Henschke 2009 [96]	0.88 (0.47–1.00)	0.50 (0.47–0.53)	1.8 (1.3–2.3)	0.25 (0.04–1.57)
The Henschke rule 2 out of 3 positive findings (H) Henschke 2009 [96]	0.63 (0.31–0.86)	0.96 (0.95–0.97)	15.5 (8.4–28.4)	0.39 (0.16–0.96)
Age >70 years (H)	0.50 (0.22–0.78)	0.96 (0.94–0.97)	11.2 (5.3–23.6)	0.52 (0.26–1.05)
Significant trauma (major in young, minor in elderly) (H)	0.25 (0.07–0.59)	0.98 (0.96–0.98)	10.0 (2.9–35.1)	0.77 (0.52–1.15)
Prolonged use of corticosteroids (H)	0.25 (0.07–0.29)	0.99 (0.99–1.00)	48.5 (11.5–204)	0.75 (0.51–1.13)
Studies supporting items of the Henschke rule				
Age >74 years (H) van den Bosch 2004 [97]	0.59 (0.48–0.69)	0.84 (0.82–0.86)	3.7 (3.0–4.5)	0.49 (0.38–0.63)
Trauma (H) Gibson 1992 [98]	1.00 (0.59–1.00)	0.51 (0.41–0.62)	2.1 (1.7–2.5)	0.00 (NA)
Trauma (H) Patrick 1983 [99]	0.80 (0.65–0.90)	0.55 (0.51–0.59)	1.8 (1.5–2.1)	0.36 (0.20–0.68)
Trauma (H)^a^ Scavone 1981 [103]	0.65 (0.44–0.83)	0.95 (0.93–0.96)	12.8 (8.6–19.2)	0.37 (0.22–0.62)
Studies not supporting items of the Henschke rule				
Trauma (H) Deyo 1986 [100]	0.36 (0.16–0.62)	0.90 (0.86–0.93)	3.4 (1.6–7.4)	0.71 (0.49–1.06)
Trauma (H) Reinus 1998 [101]	0.07 (0.02–0.18)	0.60 (0.56–0.65)	0.18 (0.07–0.5)	1.54 (1.38–1.71)
Using steroids (H) Deyo 1986 [100]	0.00 (0.00–0.23)	0.99 (0.98–1.00)	0.0 (NA)	1.01 (0.99–1.02)
Additional findings reported by more than one study				
The Roman rule 2 out of 5 positive findings: age >52 years, no leg pain, body mass index >22, no regular exercise, female gender (H) Roman 2010 [102]	0.95 (0.83–0.99)	0.34 (0.33–0.34)	1.4 (1.3–1.8)	0.16 (0.04–0.51)
Female gender (H) van den Bosch 2004 [97]	0.72 (0.62–0.81)	0.42 (0.41–0.45)	1.2 (1.1–1.5)	0.64 (0.46–0.92)
Female gender (H) Roman 2010 [102]	0.89 (0.75–0.97)	0.41 (0.38–0.44)	1.5 (1.3–1.7)	0.26 (0.10–0.65)
Neurological signs not specified (P) Gibson 1992 [98]	29 (0.04–0.71)	0.88 (0.80–0.94)	2.4 (0.67–8.7)	0.81 (0.51–1.30)
Neurological signs not specified (P) Reinus 1998 [101]	0.05 (0.01–0.15)	0.92 (0.89–0.94)	0.7 (0.2–2.2)	1.03 (0.96–1.10)
Sensory deficits not specified (P) Patrick 1983 [99]	0.03 (0.00–0.13)	0.98 (0.97–0.99)	1.4 (0.2–10.9)	0.99 (0.94–1.04)
Sensory deficits not specified (P)^a^ Scavone 1981 [103]	0.27 (0.12–0.83)	0.88 (0.85–0.90)	2.2 (1.1–4.3)	0.83 (0.66–1.05)
Motor deficits not specified (P) Patrick 1983 [99]	0.02 (0.00–0.13)	0.99 (0.98–1.00)	3.1 (0.4–27.3)	0.98 (0.94–1.03)
Motor deficits not specified (P)^a^ Scavone 1981 [103]	0.23 (0.09–0.44)	0.89 (0.87–0.91)	2.2 (1.1–4.5)	0.86 (0.70–1.06)
Deep tendon reflex abnormality not specified (P) Patrick 1983 [99]	0.08 (0.02–0.20)	0.95 (0.93–0.97)	1.5 (0.5–4.9)	0.97 (0.89–1.06)
Deep tendon reflex abnormality not specified (P)^a^ Scavone 1981 [103]	0.12 (0.02–0.30)	0.89 (0.87–0.91)	1.1 (0.4–3.2)	0.99 (0.86–1.14)
Tenderness not specified (P) Patrick 1983 [99]	0.73 (0.56–0.85)	0.59 (0.54–0.63)	1.8 (1.4–2.2)	0.47 (0.28–0.78)
Tenderness not specified (P)^a^ Scavone 1981 [103]	0.50 (0.32–0.68)	0.73 (0.70–0.76)	1.9 (1.3–2.8)	0.68 (0.46–1.00)
Spasm not specified (P) Patrick 1983 [99]	0.25 (0.13–0.41)	0.83 (0.79–0.86)	1.5 (0.83–2.6)	0.90 (0.75–1.09)
Spasm not specified (P)^a^ Scavone 1981 [103]	0.12 (0.04–0.29)	0.91 (0.89–0.93)	1.3 (0.4–3.7)	0.98 (0.85–1.12)

(?) = No original data presented to allow for calculation of CI. (---) = Calculation not possible. Calculations are based on number of patients

*H* history or questionnaire finding, *P* physical examination finding, *PVD* peripheral vascular disease, *LR* likelihood ratio, *CI* confidence interval, *NA* not applicable

^a^Values transferred from previous systematic reviews

Because of heterogeneous study populations, performance of index tests, and choice of reference standards, only descriptive statistics were used to summarize findings across studies. The diagnostic value of findings in each category is presented below.

### Intervertebral disc

A previous systematic review of clinical diagnosis of lumbar intervertebral discs (ID) has terminated the literature search at February 2006 [30], Therefore, databases were searched by the present authors from that date up to May 2015. The results of the search are presented in Additional file 7. Three studies [31–33] from the Hancock review and one study [34] from our updated search were included (Table 2).

The evidence is sufficient to constitute a Clinical Diagnostic Rule (CDR). We recommend the use of centralization of symptoms during physical examination. Two studies using strict criteria for centralization (change of pain in the furthermost whole body region) reported high levels of positive LR [32, 33], meaning that a positive test is useful for ruling in the diagnosis. One study using less strict criteria for centralization (change in any furthermost extent of pain] [31], However, a positive LR of 2.1 even in this study indicates the presence of relatively few false positive tests.

### Facet joint

A previous systematic review of clinical diagnosis of facet joints (FJ) terminated the literature search at February 2006 [30]. The current search started from that date up to May 2015. The results are presented in Additional file 7. Seven studies [32, 35–40] from the Hancock review and three studies [41–43] from our updated search were included in this review (Table 2).

The evidence is insufficient to constitute a CDR. No studies supporting Revel’s suggested rule [35] or part thereof were identified.

The only negative findings from studies with single block reference standards that appeared potentially useful for ruling out FJ pain were centralization [32, 39] and no relief with recumbency [37, 38].

### Sacroiliac joint

A previous systematic review of clinical diagnosis of sacroiliac joints (SIJ] terminated the literature search at February 2006 [30]. The current search started from that date up to May 2015. Results are presented in Additional file 7. Four studies [32, 44–46] from the Hancock review and three studies [47–49] from our updated search were included (Table 2).

The evidence is sufficient to constitute a CDR. We recommend the use of the Laslett rule [44] comprising at least 3 positive out of 5 of the following findings from physical examination: distraction, compression, thigh thrust, Gaenslen’s test, or sacral thrust.

The rule was supported by two additional studies where composites of at least 3 positive out of 5 tests resulted in high levels of positive LR [45, 48]. There is only a slight difference in tests included in the composites.

We recommend the addition of no centralization from the “Laslett composite” to the CDR as it increases the positive LR without compromising the negative LR. The value of centralization for screening out SIJ pain was supported by one more study with single block reference standards reporting an acceptable negative LR [32].

Furthermore, we recommend the use of the physical examination finding dominant pain the posterior superior iliac crest (PSIS) area. This finding was only investigated in one study using the double block standard [49]. However, the usefulness is supported by the fact that all included studies comprised patients with pain location in the PSIS area and it is a logical assumption that a strict interpretation of pain location; i.e. dominant pain in the PSIS area opposed to any level of pain, will increase the specificity of this finding.

### Disc herniation with nerve root involvement

A systematic review in the field of clinical diagnostic of disc herniation with lumbar nerve root involvement (NRI) has terminated the search of literature at October 2008 [50] and an update is in progress. [51] Therefore, no search of the literature was performed by the present authors. However, we reviewed the included studies and the reference lists of those studies for additional clinical findings. Thirteen studies [52–64] were included from the systematic review and one study was excluded due to lack of a reference standard negative population [65]. In addition, eight studies were included from the latest Cochrane review [66] and our hand search of reference lists [67–74] (Table 2). Data from original studies were reviewed and new calculations of diagnostic values were performed as appropriate.

The evidence is sufficient to constitute a CDR. We recommend initial screening by use of the straight leg raise (SLR) test in combination with the Hancock rule [52] comprising at least 3 positive out of 4 of the following findings: dermatomal pain location in concordance with a nerve root, and corresponding sensory deficit, reflex and motor weakness.

The CDR was supported by another composite [74] who reported the diagnostic value of a combination of 3 neurological signs in patients with monoradicular pain.

The value of a negative SLR test for screening out nerve root involvement was supported by the vast majority of single studies reporting acceptable levels of negative LRs regardless of level of nerve root involvement [55–58, 62–64, 71, 72].

Furthermore, we recommend the use of crossed SLR that was supported by acceptable positive LRs in the vast majority of studies [55, 58, 59, 62, 70].

The single findings included in the Hancock rule were supported by most studies reporting diagnostic value. Findings were supported by studies reporting acceptable levels of positive LRs: dermatomal S1 pain location [54], L2-L5 sensory deficits [55–57], L4 patellar reflex weakness [56, 58], S1 Achilles reflex weakness [55–58], L4 knee extension weakness [56], L5 dorsiflexion weakness of ankle and toes [55, 56, 58], or S1 plantarflexion weakness of ankle [55, 56]. One study reported acceptable level of negative LR: any nerve dermatomal pain location [53].

The diagnostic value of dermatomal pain location in the Hancock rule was supported by only one additional study and only regarding S1 distribution [54]. However, the usefulness is supported by the fact that 11 out of 14 studies included a patient population with radicular pain location, and it is a logical assumption that a strict interpretation of radicular pain; i.e. dermatomal distribution corresponding neurological findings, will increase the specificity of this finding.

### Spinal stenosis

A recently updated systematic review in the field of clinical diagnostic of lumbar spinal stenosis (SS) terminated at March 2011 [75]. Therefore, no search of the literature was performed by the present authors. Nine studies [76–84] were included from the systematic review (Table 2). Two of the nine studies included the same population [82, 84] and we chose to use values from one [82] because it reported diagnostic accuracy of questionnaire items not necessarily part of the reference standard based on physical examination and imaging. In addition, we included one study that was identified by our hand search of reference lists [85].

The evidence is sufficient to constitute a CDR. We recommend the use of the Cook rule [76] comprising at least 3 positive out of 5 of the following findings from patient history: age more than 48 years, bilateral symptoms, leg pain more than back pain, pain during walking/standing, and pain relief upon sitting (Table 2). Furthermore, we recommend the use of improved walking tolerance with the spine in flexion that was supported by two studies with acceptable levels of positive LRs [83, 85], and the patient history report of relief by forward bending that was supported by two studies with acceptable levels of positive LRs [77] or negative LRs [79].

The single findings included in the Cook rule were supported by other studies reporting diagnostic value. Some findings were supported by studies reporting high levels of positive LRs: age above 50 years [77], bilateral pain [78], severe leg pain [79], leg pain worse with walking [77, 80], pseudoclaudication [81], pain worse with standing [77], and symptoms improved when seated [79]. Other studies reported acceptable levels of negative LRs: no leg pain [77, 81], pain not worse when walking or standing [82, 83], and sitting not best posture [83].

### Spondylolisthesis

A recently updated systematic review of clinical diagnosis of lumbar spondylolisthesis terminated at March 2010 [86]. Therefore, databases were searched by the present authors from that date up to May 2015. Results of the search are presented in Additional file 8. Three studies from the systematic review [87–89] and five studies from our updated search [90–94] were included (Table 2).

The evidence is sufficient to constitute a CDR. We recommend a combination of two physical examination findings positive: intervertebral slip by inspection or palpation and segmental hypermobility by use of manual passive physiological intervertebral motion test (Table 2). Furthermore, we recommend the use of the passive lumbar extension test as a supplement for the identification of degenerative spondylolisthesis in the elderly. All tests were supported by two studies with acceptable levels of positive LRs.

### Fracture

An recently updated systematic review of the diagnosis of lumbar fracture terminated at March 2012 [95]. Therefore, no search of the literature was performed by the present authors. Eight studies from the systematic review [96–103] were included (Table 2).

The evidence is insufficient to constitute a CDR. Best evidence synthesis indicates the potential benefit of the Henschke rule [96] comprising at least 1 negative out of 3 of the following findings from patient history: findings: age >70 years, prolonged use of corticosteroids, and significant trauma (Table 2). This rule presented with the lowest negative LR meaning that when none of these findings are present, the clinician will be able to rule out a lumbar fracture with acceptable confidence.

Regardless of setting in which the studies were conducted, single studies provided inconsistent results, and the Henschke rule has not been validated in other studies.

### Myofascial pain

There is no available evidence regarding diagnostic value. We have conducted a systematic search of the literature to May 2015 revealing that studies in the field are hampered by the lack of an adequate diagnostic reference standard. The results of the search are presented in Additional file 9. It appears that clinical criteria are in fact the reference standard. Firm manual pressure applied to the muscle and elicited feedback from the patient appears to be the only means to establish the diagnosis. However, there is considerable variability of criteria used to diagnose a Myofascial Pain Syndrome [104]. The original criteria for a myofascial trigger point (TrP) originally proposed by Travell and Simons [105], have been revised based on clinical experience and results from reliability studies, but neither have been rigorously validated [104].

We suggest a composite of four minimum criteria that support the diagnosis: 1) presence of a palpable taut band within a skeletal muscle, 2) presence of a hypersensitive spot within the taut band with or without reproduction of a distinct referred pain sensation with stimulation of the spot, 3) patient recognition of the elicited pain. These criteria are based on a strict interpretation of the nine criteria currently under debate by The International Association for the Study of Pain (IASP) [106].

We have found no accepted reference standard by which a TrP can be diagnosed. However, several methods have been suggested in order to at least demonstrate construct validity of the clinical criteria. The results of our search revealed some attempts to demonstrate construct validity when TrPs were compared to electromyography [107–111], sonoelastography [112], and quantitative sensory testing [113, 114]. Methodological quality is generally low due to lack of blinding, differences in definition of active and latent TrPs, and all studies but two [108, 113] investigated the shoulder and neck region making generalizability questionable when results are transposed to the low back.

In the absence of evidence regarding diagnostic accuracy, physical examination findings should demonstrate inter-rater reliability in order to be considered clinically meaningful. Two recent systematic reviews conclude that physical examination findings cannot identify TrPs with an acceptable degree of reliability [115, 116]. However, the authors state that if diagnostic criteria were revised to include only a palpable tender spot in the muscle that when palpated reproduces the patients’ familiar pain in that spot or in a distinct pattern, then the present evidence indicates that worthwhile agreement might be achieved. This reasoning is in line with our suggestion of including three of the IASP criteria.

There are significant issues in relation to the intra- and inter-observer reliability of identifying a muscle containing a TrP, and there are no data supporting the ability of different examiners to agree on the exact location of a TrP within a specific muscle.

Taken together, no conclusions can be made based on the present evidence although our suggested criteria to be used in future diagnostic studies appear to have face validity.

### Peripheral nerve

There is no available evidence regarding diagnostic value. We have conducted a systematic search of the literature up to May 2015 revealing that all studies in the field are hampered by the lack of an adequate diagnostic reference standard. The results of the search are presented in Additional file 10. It appears that clinical criteria are in fact the reference standard. We suggest the following criteria to be used in future diagnostic studies: Patient recognition of usual lumbar or leg pain with at least two stages of sensitizing maneuvers, i.e. knee extension, ankle dorsiflexion, or neck flexion during SLR or slump test.

Although it has not been possible to report rigorous diagnostic validity of our suggested criteria, they appear to have some degree of face validity across authors. However, there is considerable variability of criteria used to diagnose increased peripheral neural mechanosensitivity [117]. Most commonly used are SLR and slump, but the interpretation of a positive test response differs. Authors may put emphasis on provocation of any lumbar or leg pain, patient recognition of their usual pain, and/or restriction of movement during testing [118].

Our search identified no studies that made comparisons between peripheral nerve mechanosensitivity testing and diagnostic procedures that appear to have the potential to be considered as reference standard (i.e. nerve conduction electrodiagnostics, ultrasound imaging, or magnetic resonance neurography]. However, our literature searches identified a number of studies attempting to demonstrate construct validity of particular aspects of the clinical representation of peripheral nerve pain.

Several studies found that reduction in range of movement (ROM] during SLR or slump as criterion for increased neural mechanosensitivity had no proven value in discriminating between patients with LBP and asymptomatic persons [119–124]. Also the hypothesis, that increased muscle tension might be responsible for the changes in ROM during SLR and slump test, has been refuted by electromyographic studies [122, 125–127]. These studies found that muscle tension is an unlikely source to ROM reduction during SLR and slump, but they did not address the main concern, that is, that any fascial network in the back and legs would be a equally plausible source of pain provocation during neural sensitizing maneuvers. Taken together, the data support the view of Shacklock [118] who claimed that reproduction of the patients usual symptoms should be an integral part of the diagnostic criteria.

In the absence of an accepted reference standard, physical examination findings should demonstrate inter-rater reliability in order to be considered clinically meaningful. Our search did not identify any reviews exploring the inter-tester reliability of SLR or slump in patients with LBP. However, we found three individual studies in which the inter-tester reliability of patient recognition of lumbar or leg pain with at least two stages of sensitizing maneuvers was investigated. In all studies, Kappa values (K] indicated substantial agreement between examiners [128]. Walsh et al.[129] reported K = 0.80 (CI 0.39–0.94) for SLR and 0.71 (CI 0.33–0.71) for Slump, Philip et al. [130] reported K = 0.89 (CI 0.81–0.97) for Slump, and Petersen et al. [12] reported K = 0.59 (CI 0.39–0.79) for SLR and Slump.

To summarize, no conclusions can be made based on the present evidence although our suggested criteria to be used in future diagnostic studies appear to have face validity and acceptable level of intertester reliability.

### Central sensitization

There is insufficient evidence to generate a diagnostic rule to identify patients with a condition characterized by “increased responsiveness of nociceptive neurons in the central nervous system to their normal or subthreshold afferent input” [131]. We have not conducted a systematic search of the literature inasmuch as studies in the field are hampered by the lack of an adequate diagnostic reference standard because the underlying mechanisms behind localized, regional and widespread pain are not fully understood [132, 133]. In the absence of anything better, we suggest the consensus-based Nijs rule to support the diagnosis of central sensitization (CS) [134].

The first step in the rule is to exclude a neuropathic pain source by use of the IASP criteria [135] and NeuPSIG guidelines [136]. The next step is to make sure that the following criterion 1 is satisfied in combination with either criterion 2 or 3:

- Criterion 1. Pain experience disproportionate to the nature and extent of injury or pathology, i.e. not sufficient evidence of injury, pathology, or objective dysfunctions capable of generating nociceptive input consistent with the patient’s severity of pain and disability.
- Criterion 2. At least one of the following patterns present:
  - bilateral pain/mirror pain (i.e., symmetrical pain pattern)
  - pain varying in (anatomical) location/travelling pain to anatomical locations unrelated to the presumed source of nociception e.g., hemilateral pain, large pain areas with non-segmental (i.e., neuroanatomically illogical) distribution
  - widespread pain (defined as pain located axially, on the left and right side of the body and both above and below the waist)
  - allodynia/hyperalgesia outside the segmental area of (presumed] nociception. These findings are based on testing of light touch by means of a swap or cold items (allodynia) as well as testing by pin prick or pressure (hyperalgesia).
- Criterion 3. Hypersensitivity of senses unrelated to the muscular system. These findings are based on a score of at least 40 on the Central Sensitization Inventory [137, 138].

Our suggested criteria are based on a consensus report by researchers from different professions [134] and are in line with other experts in neurophysiology [139–141]. Thus, although it has not been possible to report diagnostic value of the criteria, and only aspects of construct validity have been reported [142], they appear to have face validity. Results of systematic reviews are not consistent with respect to prevalence of generalized or widespread sensitization after quantitative sensory testing as stand-alone tests in patients with chronic LBP [142, 143]. However, a composite of criteria fairly similar to those of the Nijs rule for separating CS from nociceptive and peripheral neuropathic pain sources have been reported to have acceptable levels of inter-tester reliability (K = 0.77, CI 0.57–0.96) [144] and discriminative validity (positive LR 40.6, CI 20.4–80.8) [145].

Taken together, no conclusions can be made based on the present evidence although our suggested criteria to be used in future diagnostic studies appear to have face validity, and promising aspects of construct validity and level of intertester reliability has been reported.

## Discussion

We found no composites of clinical findings that were able to fully substitute for the respective reference standards. Thus, in cases where a patho-anatomical diagnosis is of crucial importance for the clinician or the patient, the patient must be referred for more sophisticated diagnostic procedures, which may include high tech imaging or minimally invasive, controlled and guided injection procedures.

### Intervertebral disc

Our recommendation for the disc CDR is strong due to risks of partial verification bias in only one [32] of the three studies investigating the finding of centralization. In all studies, a high risk of selection bias is present, because they included patients from secondary care referred for diagnostic invasive procedures. Consequently, the studies are likely to overestimate the diagnostic gain of using the CDR in comparison to primary care settings where the prevalence is somewhat lower.

In addition to the discography studies, our search identified two studies reporting the diagnostic value of centralization for identifying patients with MRI findings of extruded or sequestrated discs [146, 147] Results of these studies were not in concordance and warrant further investigation.

### Facet joint

It was not possible to constitute a CDR for the identification of painful FJ. Double block procedure in joint space or at nerve supply was judged to be acceptable as reference standard when at least one of the following criteria were satisfied: a positive controlled block, i.e. the anesthetic block definitely reduced the pain from the injected joint, where as a block in a non-painful joint had no marked effect on pain, a positive confirmatory block, the anesthetic block definitely reduced the pain from the injected joint at two separate occasions 1 to 2 weeks apart, or a positive comparative dual block, i.e. a short- followed by a long lasting anesthetic significantly reduced pain in the predicted time periods [148].

The only negative findings from studies with single block reference standards that supported single tests of the Revel rule for ruling out FJ pain was no relief with recumbency [37, 38]. However, the quality of evidence for this finding was downgraded due to serious risk of test review bias in both studies.

We found two additional single block studies investigating diagnostic value of non-centralization using a single block reference standard [32, 39]. Both studies reported acceptable levels of sensitivity (0.96 and 0.97 respectively) and negative LRs (0.22 and 0.28 respectively). However, the quality of evidence for this finding was downgraded due to risk of partial- or differential bias in the two studies. Although validated with only a single block reference standard, a finding of centralization might have preliminary merit for ruling out a symptomatic facet joint because there is no point in giving patients with a negative screening block a second block, even if the second block was positive the same conclusion is reached, non-FJ pain. The same reasoning applies to the value of no relief in recumbency.

The results regarding no relief with recumbency and non-centralization appear promising, but they need verification in future studies.

It is unclear whether the three studies by Manchikanti et al. [35, 36, 41] might include the same populations. However, this issue would have no influence on the conclusion.

### Sacroiliac joint

Our recommendation for the SIJ CDR is strong. Only one out of three studies supporting the diagnostic value of the composite of tests displayed risk of differential bias [44]. In all studies, however, a high risk of selection bias is present, because they included patients from secondary or tertiary care referred for diagnostic invasive procedures. The CDR is supported by an additional two out of three studies where composites of at least 3 positive out of 5 tests resulted in high levels of positive LRs [45, 48]. Although the content of the composites are comparable there is a slight difference in the use Patrick’s PABER test and Mennell’s test. The fact that one study did not support the rule [47], might be explained by the fact that the double block were performed only 30 min apart, which increases the risk of false positive findings. Furthermore, the quality of this study suffered from the risk of test review bias.

The recommendation of no centralization during physical examination was weak based on two studies [32, 44]. One of those was reporting an acceptable level of negative LR for centralization using a single block reference standard, making non-centralization useful for ruling out a symptomatic SIJ [32]. However, both studies suffered from risk of partial verification bias leading to a downgrading of the quality of evidence.

We found two additional studies investigating diagnostic value of SIJ area pointing, without indication of whether or not the pain was dominant, using insufficient reference standard in terms of a single or periarticular SIJ blocks [46, 149]. The results were not in concordance and warrant further investigation.

### Nerve root involvement

The strength of our recommendation for the CDR is weak based on mediocre methodological quality in most of the studies. Studies revealed serious risk of bias in relation to differential verification, incorporation, or test review.

The studies included used surgical or imaging findings as a reference standard. We found no differences in diagnostic values when results from surgical and imaging studies were compared, which indicates that the findings are similar across reference standards used. Readers, interesting in results from pooling of studies exclusively using surgery as reference standard, are referred to the most recent systematic reviews [50, 66].

The reference standards have an influence on the diagnostic value of index tests. Studies using surgery means that results were obtained in a patient population with high prevalence of severe disc herniations, and thus results cannot be generalized to primary care populations where prevalence is much lower. Studies using imaging may display prevalence more like what is found in primary care, however at the expense of more false positive findings [150]. Consequently, uncertainty remains as to the generalizability of the results in primary care settings. Only two studies [53] and [68] included patients representative of those seen in primary care.

As suggested by others [66] we have tried to increase the performance of tests in clinical practice by recommending a CDR using a combination of tests with high levels of sensitivity and specificity. Other combinations of tests have been suggested [53, 69, 72, 151], but these are not summarized in the format of CDRs and they are not supported as well by single studies as the Hancock rule.

When possible, we chose to report one level disc or nerve root as reference standard in order to reduce the number of false positives due to noise from other non-relevant levels. This choice reflects the clinical reasoning process in daily practice. The clinician needs to compare dermatomal pain distribution with corresponding motor or reflex weakness in order to make a meaningful diagnostic pattern.

### Spinal stenosis

The strength of our recommendation for the CDR is weak, based on low methodological quality of studies. Many of the quality items revealed serious risks of bias. First, the index test was part of the reference standard (incorporation bias) in all studies resulting in a high risk of overestimation of the diagnostic value of findings. Most studies used expert opinion based on a combination of physical examination findings and imaging even though data suggest that imaging is probably not sufficient as a reference standard in comparison with surgical findings [150]. Only two studies used surgical verification of diagnosis as part of the reference standard [77, 78]. Second, the majority of studies had problematic reporting of blinding (test review bias) i.e. whether the reference standard result was interpreted blind to those of the index test and vice versa [76–78, 82, 83, 85]. Third, all studies included patients from secondary or tertiary settings with a high prevalence of patients with SS. Consequently, there is a high risk of selection bias that is likely to overestimate the diagnostic gain of using the CDR in comparison to primary care settings where the prevalence is dramatically lower.

### Spondylolisthesis

The strength of our recommendation for the CDR is strong based on the methodological quality of studies. Although several of the studies displayed risk of disease progression bias and poor description of index tests, the quality items reveal serious risks of bias in few cases [90, 94].

In the present review, functional dynamic radiographs were accepted to identify segmental instability if index tests were pain provocation or movement tests and plain static radiographs if index tests were palpation of slip.

Flexion-extension functional radiographs are considered the “gold standard” in degenerative spondylolisthesis, and a disc angle change >10° or change in translation > 3 mm are generally used as cut-offs [152]. Plain radiographs with lateral views are useful in the initial investigation of isthmic spondylolisthesis [153]. A slip of > 3 mm has been suggested as cut-off [154], but the literature is lacking as to what degree of slip is significant [153]. Instead, the descriptive Meyerding classification [154] is often reported.

All studies used a definition of spondylolisthesis similar to the above, except Abbott et al. [88] that used a cut-off of 2 standard deviations beyond the mean of a sample of pain free individuals.

Even though the positive LRs across single studies are only of moderate levels, the magnitude of LRs will probably rise to a level sufficient to be useful in clinical practice when they are used in combination.

All studies, except one [88] were performed in tertiary settings resulting in high risk of selection bias that is likely to overestimate the diagnostic gain of using the CDR when applied to primary care.

### Fracture

It was not possible to constitute a CDR for the identification of a painful fracture. Results of single studies were not in concurrence and the majority of studies had serious risks of bias with respect to differential verification, test review, and uninterpretable results/withdrawals.

A symptomatic fracture is considered a ‘red flag’ warranting referral to secondary care. Consequently we have emphasized findings that are able to exclude patients with this condition.

The Henschke rule [96] has the potential to be a useful screening tool in primary care. However, the results need confirmation in future studies as the results of the only other primary care study included in this review were not in concordance [100]. Overall, the results from these two studies did not differ markedly from the rest.

Trauma (major in young persons and minor in the elderly] is a highly plausible mechanism that can lead to fracture and a highly increased prevalence of osteoporotic fractures are seen in patients, mainly female, with age above 75 years [97]. Both of these features contribute to the diagnostic value of the rule although not validated as stand-alone findings.

The inconsistency of results may be influenced by the method of imaging. Radiography was used in all studies with the addition of CT-scan in only one study [102]. No study used MRI. Radiographs may be adequately sensitive, but their ability to distinguish acute from chronic fractures is poor. MRI is more specific because it identifies marrow edema or an associated hematoma, which may indicate a symptomatic fracture [155].

### Myofascial pain

The suggested criteria should be regarded as the first step in defining a common set of diagnostic criteria for selection of patients to be included in future reliability and validity studies.

Our literature searches identified a number of studies attempting to demonstrate construct validity, but we did not perform a systematic search for additional studies in reference lists. Therefore, the included studies must be regarded as important examples of attempts of validation rather than a systematic review of this type of literature. The studies used TrPs found by manual palpation as the reference standard, meaning that the purpose of these studies were to identify the underlying physiological mechanisms behind the presence of TrPs rather than a diagnostic validation of palpation findings. Several hypothetical theories have been suggested in order to explain the formation and persistence of TrPs [156].

It is a matter of controversy whether TrPs should be regarded as stand-alone entities that are a primary pain source or whether they are secondary to other painful disorders [106, 157]. Consequently, a myofascial pain syndrome may coexist with several other syndromes in our proposed classification system. It is essential to exclude underlying disorders capable of causing reproduction of a referred pain sensation with stimulation of a hypersensitive spot in the muscle before a conclusion can be made as to whether the myofascial TrP is the dominant source of the patient’s pain.

### Peripheral nerve

While diagnostic value of the SLR and slump is demonstrated in patients with lumbar radiculopathy, the value in relation to painful peripheral nerve tissue is unknown. Our search did not identify any studies investigating the ability of these tests to discriminate patients with peripheral nerve pain from other competing disorders. The suggested criteria should be regarded as an attempt to define a common set of diagnostic criteria for selection of patients to be included in future validity studies.

The spread of sensitizing effects along the nerve is a plausible explanation for why movement of a distant body part can change sensory responses. However, it has been argued that the fascial network in the back and legs and may account for positive findings in terms of pain and limited range of movement during SLR and slump test [127, 158]. Therefore, structural differentiation between neural tissues as opposed to musculoskeletal connective tissues has been proposed. When lumbar or leg pain increase during the SLR test with dorsiflexion of the ankle or flexion of the neck, a neural pain source is alleged to be identified [118]. Likewise, regarding the slump test, with the addition that the pain decrease with the release of neck flexion [118, 159]. Our search of the literature did not identify any studies that specifically tested this hypothesis.

In line with other authors [160, 161], we suggest the term “Increased neural mechanosensitivity” to describe a condition where the patient’s usual pain is reproduced by sensitizing maneuvers. Increased neural mechanosensitivity has been given several other labels, i.e. adverse neural tension, neurodynamics, and neural tension dysfunction [118, 160].

The issues discussed in the myofascial pain section above, concerning coexistence with several other syndromes in our proposed classification system, apply to peripheral nerve as a pain source as well.

### Central sensitization

Although the Nijs rule is the result of a consensus process, caution is warranted because the participating experts are a selective sample within the field of neuroscience. Therefore, the suggested criteria should be regarded as an attempt to define a common set of diagnostic criteria for selection of patients to be included in future validity studies. A possible use of the Nijs rule in clinical practice has been exemplified in a recent paper [162].

CS might be explained by an amplification of neural signaling within the central nervous system that elicits pain hypersensitivity” [139] However, controversy exists as to the nature of CS and whether it is possible to identify this condition in clinical practice [140, 163].

The pathophysiological mechanisms are not fully understood, but there is increasing evidence that CS and chronic widespread musculoskeletal pain is associated with plasticity changes in of the central nervous system leading to hypersensitivity that can explain the clinical findings in chronic widespread LBP [133, 139, 141]. The main clinical manifestations are widespread lowered pain thresholds, exaggerated pain response to stimuli, and enlargement of pain referral areas. Most studies in the field have used clinical manifestations as the reference standard, meaning that the purpose of these studies were to identify the underlying physiology behind the presence of CS and widespread pain rather than a diagnostic validation of clinical findings.

In patients with chronic LBP it has been reported that 25–38% develop chronic widespread pain [164–166], and the condition is closely associated with systemic co-morbidity and psychological disorders [167].

In our opinion, the suggested rule is useful for increasing the likelihood of identifying patients with CS in primary care. Central sensitization may coexist with other structure-specific syndromes in our diagnostic classification system because it is generally recognized that there is a structural pain generator behind initial nociception and peripheral sensitization involved [132]. However, we would not expect a patient with CS to fit any of the clinical patterns of specific pain producing structures in the classification system. In order to choose the best treatment strategy, the clinician has to make a decision as to which pain sources are the dominant in the individual patient with LBP [140, 163].

### Reference standards

At the present time is seems obvious that there are no ‘gold’ standards, either in the form of clinical tests, high tech imaging or other procedures. What is available are *reference* standards that, while not perfect, are appropriate and quite adequate for the majority of patients, and for use as comparators with clinical tests in diagnostic accuracy studies. The diagnostic utility of discography and FJ or SIJ blocks is a matter of controversy. Some consensus reports do not support the use of these procedures due to insufficient evidence of validity [168], the main problem being the absence of gold standards for identifying a “true” pain source. In this review we have tried to reduce the possible false positive rate by using the strictest available criteria for the reference standards as a requirement for inclusion of studies.

What is apparent from our systematic review is that there generally is sufficient published data that can form a framework for an intelligent use of clinical examination procedures and more expensive and invasive diagnostic investigations when required. Diagnosis of the source and cause of presenting back pain remains a challenge, and only further high quality research will improve certainty for clinicians and patients alike.

It is true that for a large proportion of patients in the acute or subacute phase, an accurate patho-anatomic diagnosis is not required, even though possible with some degree of confidence. However for patients whose symptoms are not improving after several months, the need for a more precise diagnosis becomes increasingly valuable as a guide to more effective and targeted management. To this extent, the recommendations from this systematic review might be helpful, in that patient selection for expensive high tech imaging and minimally invasive diagnostic injection procedures is facilitated, with consequent better utilization of resources.

### Implication for practice

Our recommendations are based on considerations of the consequences of false positives and false negatives. In most diagnoses, we put the most emphasis on tests with high specificity indicating few false positives and positive LRs to indicate the ratio of true positive tests results above the false positives. The consequence is that the clinician will be quite certain that a patient would actually have the disorder if the reference standard procedure were to be performed. Often, high specificity is a trade off at the expense of low sensitivity, meaning that a substantial proportion of patients with the disorders are not identified, and remain unclassified. However, the consequences in primary care are not serious inasmuch as the patient remains in the category of non-specific LBP. In daily clinical practice, referral to further diagnostics most often depends on assessment of red flags, severity of symptoms and functional limitations rather than diagnostic classification.

Only in cases where an undiagnosed spinal fracture is present, do primary treatment methods have potential to harm the patient if unidentified. Consequently, we have prioritized the recommendation of tests with a high sensitivity and low negative LRs in this diagnosis.

For the clinician, the diagnostic considerations do not stop here. The diagnostic certainty that a positive test will identify a pathological disorder is dependent on the prevalence of the disorder. Prevalence of categories like nerve root involvement, spinal stenosis, spondylolisthesis, and fracture are generally much lower in primary care settings than in secondary or tertiary settings of the vast majority of diagnostic studies. This means that the diagnostic accuracy of a positive test is likely much lower when the index tests are applied to primary care settings. For example, the pre-test probability of having a symptomatic spinal stenosis in primary care is estimated to be only 3% [168]. By use of the Cook rule, the posttest probability will rise to 7%. When improved walking tolerance with the spine in flexion or patient history report of relief by forward bending are added to the rule we would expect the post-test probability to rise further. By means of the LRs presented in this review, the clinician can use Fagan’s nomogram [169] as a graphical tool for estimating how much the result on a diagnostic test changes the probability that a patient has the disorder in question.

In daily practice, it is unlikely that clinicians make conclusions based on a single finding. This practice is supported by our results that generally provide the most promising accuracy in diagnosis in which a composite of findings can be identified. Some studies do report diagnostic accuracy of test combinations and clusters, but this does not totally reflect the reasoning process of expert clinicians. Clinicians do not use individual tests or clusters of tests out of context from the total clinical picture. Sometimes pattern recognition is used, and sometimes a sequential, algorithmic or staged approach is used. Another way to utilize multiple test results is to consider the probability of specific disorders based on prevalence within a defined group or subgroup. Prevalence is equal to pre-test probability so the probability of any given disorder is equivalent to its prevalence in any given setting. The process of progressively reducing the size of the group labelled as ‘non-specific’, by abstracting out those cases with very high probability of a known condition, may be called ‘Diagnosis by Subtraction’.

### Diagnosis by subtraction

To illustrate, assume for this current purpose, that in a specific setting, the prevalence of ‘centralizers’ is 0.5 or 50%. The high specificity of this clinical finding to discogenic pain confirmed by discography indicates that these patients do not have ‘non-specific’ back pain but a ‘specific’ anatomical source of pain [33]. Whatever the prevalence of the remaining possible causes of pain in the whole group, it is twice as high in the ‘non-centralizer’ group. Thus the probability that a non-centralizer has of having, say sacroiliac joint pain or facet joint pain, is doubled. This review has shown that certain CDRs have high specificity for sacroiliac joint pain, spondylolisthesis, disc herniation with nerve root involvement, and spinal stenosis. If we sequentially subtract those cases satisfying the CDR’s for these conditions, the prevalence / probability of other conditions being the cause of pain progressively rises as the size of the non-specific low back pain category reduces.

### Limitations of this review

One of the main limitations in this review is that the search of the literature was not updated to year 2015 in all diagnostic categories. Due to limited resources, this has not been possible for the present authors. If an existing review fulfilled the criteria of being current, relevant, and of high-quality, then we chose to use our resources to conduct systematic searches within fields where recent reviews had not been published.

The vast majority of patients is most likely not representative of those that present for treatment in primary care. Almost all patients were preselected having a referral to specialist centers for specific diagnostic evaluation making them likely to have the target disorder in question.

Although some of the included reviews have used a QUADAS score of 10/14 as a marker for high versus low quality studies, we agree with the developers of the tool that no meaningful cut off exists [170].

It is our judgment that pooling of data was not feasible due to great variability across studies: The patient characteristics and prevalence of the target disorders varied considerably, the same reference standard was seldom used across studies, definition of a positive reference standard was not often specified, and execution of index tests was likely to vary among studies. Though it is tempting to pool data and perform a meta-analysis, we chose not to do this since in our opinion, pooling systematically homogenizes studies that are in fact acknowledged as heterogeneous. We chose to put emphasis on the results of those studies that had satisfactory quality assessments, and seemed to be closest in context to the environment this classification targets i.e. primary care.

## Conclusions

In some diagnostic categories we have sufficient evidence to suggest a CDR. In others, we have only preliminary evidence that needs testing in future studies. The use of single clinical tests appears to be less useful than clusters of tests which is more closely in line with clinical decision making.

**Fig. 1:**
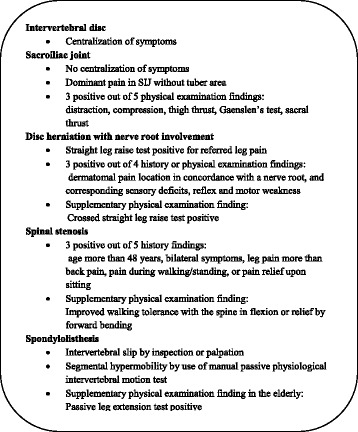
Promising Clinical Diagnostic Rules based on best-evidence

With respect to clinical diagnostic of symptomatic intervertebral disc, sacroiliac joint, spondylolisthesis, disc herniation with nerve root involvement, and spinal stenosis, we were able to construct promising CDRs (see Fig. 1]. However, the accuracy of these findings in a primary care setting has yet to be confirmed.

## Additional files

Additional file 1: Search strategy for disc, sacroiliac joint, and facet joint. (DOCX 25 kb)

Additional file 2: Search strategy for spondylolisthesis. (DOCX 24 kb)

Additional file 3: Search strategy for myofascial pain. (DOCX 19 kb)

Additional file 4: Search strategy for peripheral nerve pain. (DOCX 22 kb)

Additional file 5: Characteristics of the included studies. (DOCX 37 kb)

Additional file 6: Quality assessment of included studies. (DOCX 35 kb)

Additional file 7: Flow chart for selection of disc, sacroiliac joint and facet joint articles. (DOCX 12 kb)

Additional file 8: Flow chart for selection of spondylolisthesis articles. (DOCX 12 kb)

Additional file 9: Flow chart for selection of myofascial pain articles. (DOCX 12 kb)

Additional file 10: Flow chart for selection of nerve pain articles. (DOCX 12 kb)

## Abbreviations

CDR: Clinical diagnostic rule
CS: Central sensitization
CT: X-ray computed tomography
FJ: Facet joint
ID: Lumbar intervertebral disc
LBP: Low back pain
LR: Likelihood ratio
MRI: Magnetic resonance imaging
NRI: Lumbar nerve root involvement
QUADAS: Quality Assessment of Diagnostic Accuracy Studies
ROM: Range of movement
SIJ: Sacroiliac joint
SLR: Straight leg raise
SS: Lumbar spinal stenosis
TrP: Myofascial trigger point
