# Supplementary material for: Clinical classification in low back pain: best-evidence diagnostic rules based on systematic reviews
Source: BMC Musculoskelet Disord. 2017 May 12;18:188. doi: 10.1186/s12891-017-1549-6 (PMC5429540; doi:10.1186/s12891-017-1549-6)
Supplement: Supplementary file 1 — Search strategy for disc, sacroiliac joint, and facet joint. (DOCX 25 kb) [file 12891_2017_1549_MOESM1_ESM.docx]

Additional file 1a. PubMed search strategy for disc, sacroiliac joint, and facet joint.

1. (sensitivity AND specificity)[All Fields]

2. specificity[All Fields]

3. accuracy[All Fields]

4. screening[All Fields]

5. false negative[All Fields]

6. false positive[All Fields]

7. predictive value[All Fields]

8. predictive value of tests[All Fields]

9. reference value[All Fields]11.

10. roc[All Fields]

11. roc analysis[All Fields]

12. roc area[All Fields]

13. roc auc[All Fields]

14. roc characteristics[All Fields]

15. roc curve*[All Fields]

16. roc curve method[All Fields]

17. roc estimated[All Fields]

18. roc evaluation[All Fields]

19. likelihood ratio[All Fields]

20. diagnostic procedure[All Fields]

21. Diagnostic Tests, Routine[Mesh:noexp]

22. diagnostic tests[All Fields]

23. Low Back Pain/diagnosis[Mesh:noexp]

24. diagnos*[Title/Abstract]

25. Pain/diagnosis[Mesh:noexp]

26. prognosis[All Fields]

27. 1 or 2 or 3 or 4 or 5 or 6 or 7 or 8 or 9 or 10 or 11or 12 or 13 or 14 or 15 or 16 or 17 or 18 or 19 or 20 or 21 or 22 or 23 or 24 or 25 or 26

28 medical history taking[All Fields]

29. physical examination[All Fields]

30. Physical Examination[Mesh ]

31. Radiography[Mesh]

32. radiography[All Fields]

33. x-ray[All Fields]

34. X-Rays[Mesh]

35. Low Back Pain/radiography[Mesh:noexp]

36. Back Pain/radiography[Mesh:noexp]

37. Spine/radiography[Mesh:noexp]

38. Spinal Diseases/radiography[Mesh:noexp]

39. Lumbar Vertebrae/radiography[Mesh:noexp]

40. Intervertebral Disc/radiography[Mesh:noexp]

41. Intervertebral Disc/radionuclide imaging[Mesh:noexp]

42. Magnetic Resonance Imaging[Mesh]

43. magnetic resonance imaging[All Fields]

44. computed tomography[All Fields]

45. Tomography, X-Ray Computed[Mesh]

46. Nuclear Medicine[Mesh]

47. nuclear medicineTomography, Emission-Computed, Single-Photon[Mesh]

48. single photon emission computed tomography[All Fields]

49. Back Pain/radionuclide imaging[Mesh:noexp]

50. Low Back Pain/radionuclide imaging[Mesh:noexp]

51. Spine/radionuclide imaging[Mesh:noexp]

52. Spinal Diseases/radionuclide imaging[Mesh:noexp]

53. Lumbar Vertebrae/radionuclide imaging[Mesh:noexp]

54. radionuclide imaging[All Fields]

55. Radionuclide Imaging[Mesh]

56. scintigraphy[All Fields]

57. bone scan[All Fields]

58. Questionnaires[Mesh]

59. questionnaires[All Fields]

60. clinical history[All Fields]

61. Injections, Spinal[Mesh]

62. discography[All Fields]

63. thermography[All Fields]

64. pain provocation test*[All Fields]

65. Injections, Intra-Articular[Mesh]

66. Nerve Block[Mesh:noexp]

67. nerve block[All Fields]

68. diagnostic blocks[All Fields]

69. 28 or 29 or 30 or 31 or 32 or 33 or 34 or 35 or 36 or 37or 38 or 39 or 40 or 41 or 42 or 43 or 44 or 45 or 46 or 47 or 48 or 49 or 50 or 51 or 52 or 53 or 54 or 55 or 56 or 57 or 58 or 59 or 60 or 61 or 62 or 63 or 64 or 65 or 66 or 67 or 68

70. disc*[Title/Abstract]

71. Intervertebral Disc[Mesh]

72. disk*[Title/Abstract]

73. intervertebral disc[All Fields]

74. sacroiliac[All Fields]

75. Sacroiliac Joint[Mesh:noexp]

76. SIJ[All Fields]

77. zygapophyseal joint*[MeSH Terms]

78. facet joint*[Title/Abstract]

79. 70 or 71 or 72 or 73 or 74 or 75 or 76 or 77 or 78

80. 27 and 69

81. 79 and 80

Filters: Publication date from 2006/01/03 to 2015/04/28

Additional file 1b. EMBASE search strategy for disc, sacroiliac joint, and facet joint.

1. (sensitivity AND specificity).mp.

2. specificity.mp.

3. accuracy .mp.

4. screening.mp.

5. false negative.mp.

6. false positive.mp.

7. predictive value.mp.

8. predictive value of tests.mp.

9. reference value.mp.11.

10. roc.mp.

11. roc analysis.mp.

12. roc area.mp.

13. roc auc.mp.

14. roc characteristics.mp.

15. roc curve*.mp.

16. roc curve method.mp.

17. roc estimated.mp.

18. roc evaluation.mp.

19. likelihood ratio.mp.

20. diagnostic procedure.mp.

21. Low Back Pain/di

22. diagnos*. ti,ab

23. Pain/di

24. prognosis.mp.

25. 1 or 2 or 3 or 4 or 5 or 6 or 7 or 8 or 9 or 10 or 11or 12 or 13 or 14 or 15 or 16 or 17 or 18 or 19 or 20 or 21 or 22 or 23 or 24

26. medical history taking.mp.

27. physical examination.mp.

28. Physical Examination.mp. or exp Physical Examination/

29. exp Radiography

30. x-ray.mp. or exp X-ray/

31. Low Back Pain/

32. Back Pain/

33. Spine/

34. Spinal Diseases/

35. Lumbar Vertebrae/

36. Intervertebral Disc/

37. magnetic resonance imaging.mp. or exp Magnetic Imaging/

38. exp Tomography, X-ray Computed/ or computed tomography.mp.

39. nuclear medicine.mp. or exp Nuclear Medicine/

40. single photon emission computed tomography.mp. or exp Tomography, Emission-Computed, Single-Photon/

41. radionuclide imaging.mp. or exp Radionuclide Imaging/

42. scintigraphy.mp.

43. bone scan.mp.

44. questionnaires.mp. or exp Questionnaires/

45. clinical history.mp.

46. INJECTIONS, SPINAL/

47. diagnostic test.mp. or Diagnostic Tests, Routine/

48. discography.mp.

49. thermography.mp.

50. pain provocation test*.mp.

51. exp INJECTIONS, INTRA-ARTICULAR/ or exp INJECTIONS, SPINAL/

52. nerve block.mp. or Nerve Block/

53. diagnostic blocks.mp.

54. 26 or 27 or 28 or 29 or 30 or 31 or 32 or 33 or 34 or 35 or 36 or 37or 38 or 39 or 40 or 41 or 42 or 43 or 44 or 45 or 46 or 47 or 48 or 49 or 50 or 51 or 52 or 53

55. disc.mp.

56. discs.mp.

57. disk*.mp.

58. exp Intervertebral Disk/

59. intervertebral disc.mp.

60. sacroiliac.mp.

61. facet joint*.mp.

62. zygapophyseal joint/

63. 55 or 56 or 57 or 58 or 59 or 60 or 61 or 62

64. 25 and 54

65. 63 and 64

Filters: Publication date from 2006/01/03 to 2015/04/28

Additional file 1c. CINAHL search strategy for disc, sacroiliac joint, and facet joint.

| 1. | "sensitivity" AND "specificity" |
| --- | --- |
| 2. | "specificity" |
| 3. | "screening" |
| 4. | "false negative" |
| 5. | "false positive" |
| 6. | "accuracy" |
| 7. | "predictive value" |
| 8. | "predictive value of tests" |
| 9. | "reference value" |
| 10. | "roc" |
| 11. | "roc analysis" |
| 12. | "roc area" |
| 13. | "roc auc" |
| 14. | "roc characteristics" |
| 15. | "roc curve" |
| 16. | "roc curve method" |
| 17. | "roc curves" |
| 18. | "roc estimated" |
| 19. | "roc evaluation" |
| 20. | "likelihood ratio" |
| 21. | "diagnostic procedure" |
| 22. | (MH "Low Back Pain/DI") |
| 23. | "diagnos*" |
| 24. | (MH "Pain/DI") |
| 25. | "prognosis" |
| 26. | (1 OR 2 OR 3 OR 4 OR 5 OR 6 OR 7 OR 8 OR 9 OR 10 OR 11 OR 12 OR 13 OR 14 OR 15 OR 16 OR 17 OR 18 OR 19 OR 20 OR 21 OR 22 OR 23 OR 24 OR 25) |
| 27. | "medical history taking" |
| 28. | (MH "Physical Examination+") OR "physical examination" |
| 29. | (MH "Radiography+") OR "radiography" |
| 30. | (MH "X-Rays") OR "x-ray" |
|  |  |
|  |  |
| 31. | (MH "Low Back Pain/RA") |
| 32. | (MH "Back Pain/RA") |
| 33. | (MH "Spine/RA") |
| 34. | (MH "Spinal Diseases/RA") |
| 35. | (MH "Lumbar Vertebrae/RA") |
| 36. | (MH "Intervertebral Disk/RA") |
| 37. | (MH "Intervertebral Disk") |
|  |  |
| 38. | (MH "Magnetic Resonance Imaging+") OR "magnetic resonance imaging" |
| 39. | (MH "Tomography, X-Ray Computed+") OR "computed tomography" |
| 40. | (MH "Nuclear Medicine") OR "nuclear medicine" |
| 41. | (MH "Tomography, Emission-Computed, Single-Photon+") OR "single photon emission computed tomography" |
| 42. | (MH "Back Pain") |
| 43. | (MH "Low Back Pain") |
| 44. | (MH "Spine") |
| 45. | (MH "Spinal Diseases") |
| 46. | (MH "Lumbar Vertebrae") |
| 47. | (MH "Radionuclide Imaging+") OR "radionuclide imaging" |
| 48. | "scintigraphy" |
| 49. | "bone scan" |
| 50. | (MH "Questionnaires+") OR "questionnaires" |
| 51. | "clinical history" |
| 52. | (MH "Injections, Intraspinal") |
| 53. | (MH "Diagnostic Tests, Routine") OR "diagnostic tests" |
| 54. | "discography" |
| 55. | "diskography" |
| 56. | "thermography" |
| 57. | "pain provocation test*" |
| 58. | (MH "Injections, Intraspinal+") OR MH "Injections, Intraarticular" |
| 59. | (MH "Nerve Block") OR "nerve block" |
| 60. | "diagnostic blocks" |
| 61. | (S27 OR 28 OR 29 OR 30 OR 31 OR 32 OR 33 OR 34 OR 35 OR 36 OR 37 OR 38 OR 39 OR 40 OR 41 OR 42 OR 43 OR 44 OR 45 OR 46 OR 47 OR 48 OR 49 OR 50 OR 51 OR 52 OR 53 OR 54 OR 55 OR 56 OR 57 OR 58 OR 59 OR 60) |
| 62. | "disc" |
| 63. | "discs" |
| 64. | "disk*" |
| 65. | (MH "Intervertebral Disk") |
| 66. | "intervertebral disc" |
| 67. | (MH "Sacroiliac Joint") OR "SIJ" |
|  |  |
|  |  |
| 68. | "sacroiliac" |
| 69. | (MH “zygapophyseal joint”) |
| 70. | "facet joint" |
| 71. | 62 OR 63 OR 64 OR 65 OR 66 OR 67 OR 68 OR 69 OR 70 |
| 72. | 26 AND 61 AND 71  Filters: Publication date from 2006/01/03 to 2015/04/28 |
|  |  |
