# Supplementary material for: Clinical classification in low back pain: best-evidence diagnostic rules based on systematic reviews
Source: BMC Musculoskelet Disord. 2017 May 12;18:188. doi: 10.1186/s12891-017-1549-6 (PMC5429540; doi:10.1186/s12891-017-1549-6)
Supplement: Supplementary file 2 — Search strategy for spondylolisthesis. (DOCX 24 kb) [file 12891_2017_1549_MOESM2_ESM.docx]

Additional file 2a. PubMed search strategy for spondylolisthesis.

1. (sensitivity AND specificity)[All Fields]

2. specificity[All Fields]

3. accuracy[All Fields]

4. screening[All Fields]

5. false negative[All Fields]

6. false positive[All Fields]

7. predictive value[All Fields]

8. predictive value of tests[All Fields]

9. reference value[All Fields]11.

10. roc[All Fields]

11. roc analysis[All Fields]

12. roc area[All Fields]

13. roc auc[All Fields]

14. roc characteristics[All Fields]

15. roc curve*[All Fields]

16. roc curve method[All Fields]

17. roc estimated[All Fields]

18. roc evaluation[All Fields]

19. likelihood ratio[All Fields]

20. diagnostic procedure[All Fields]

21. Low Back Pain/diagnosis[Mesh:noexp]

22. diagnos*[Title/Abstract]

23. Pain/diagnosis[Mesh:noexp]

24. prognosis[All Fields]

25. 1 or 2 or 3 or 4 or 5 or 6 or 7 or 8 or 9 or 10 or 11or 12 or 13 or 14 or 15 or 16 or 17 or 18 or 19 or 20 or 21 or 22 or 23 or 24

26. medical history taking[All Fields]

27. physical examination[All Fields]

28. Physical Examination[Mesh ]

29. Radiography[Mesh]

30. radiography[All Fields]

31. x-ray[All Fields]

32. X-Rays[Mesh]

33. Low Back Pain/radiography[Mesh:noexp]

34. Back Pain/radiography[Mesh:noexp]

35. Spine/radiography[Mesh:noexp]

36. Spinal Diseases/radiography[Mesh:noexp]

37. Lumbar Vertebrae/radiography[Mesh:noexp]

38. Magnetic Resonance Imaging[Mesh]

39. magnetic resonance imaging[All Fields]

40. computed tomography[All Fields]

41. Tomography, X-Ray Computed[Mesh]

42. Nuclear Medicine[Mesh]

43. nuclear medicine[All Fields]

44. mography, Emission-Computed, Single-Photon[Mesh]

45. single photon emission computed tomography[All Fields]

46. Back Pain/radionuclide imaging[Mesh:noexp]

47. Low Back Pain/radionuclide imaging[Mesh:noexp]

48. Spine/radionuclide imaging[Mesh:noexp]

49. Spinal Diseases/radionuclide imaging[Mesh:noexp]

50. Lumbar Vertebrae/radionuclide imaging[Mesh:noexp]

51. radionuclide imaging[All Fields]

52. Radionuclide Imaging[Mesh]

53. scintigraphy[All Fields]

54. bone scan[All Fields]

55. Questionnaires[Mesh]

56. questionnaires[All Fields]

57. clinical history[All Fields]

58. Diagnostic Tests, Routine[Mesh:noexp]

59. diagnostic tests[All Fields]

60. pain provocation test*[All Fields]

61. 26 or 27 or 28 or 29 or 30 or 31 or 32 or 33 or 34 or 35 or 36 or 37or 38 or 39 or 40 or 41 or 42 or 43 or 44 or 45 or 46 or 47 or 48 or 49 or 50 or 51 or 52 or 53 or 54 or 55 or 56 or 57 or 58 or 59 or 60

52. spondylolisthesis

53. spondylosis

54. anterolisthesis

55. posterolisthesis

56. instability,joint [Mesh terms]

57. 52 or 53 or 54 or 55 or 56

58. 25 and 61

59. 57 and 58

Filters: Publication date from 2010/03/01 to 2015/05/06

Additional file 2b. EMBASE search strategy for spondylolisthesis.

1. (sensitivity AND specificity).mp.

2. specificity.mp.

3. accuracy .mp.

4. screening.mp.

5. false negative.mp.

6. false positive.mp.

7. predictive value.mp.

8. predictive value of tests.mp.

9. reference value.mp.11.

10. roc.mp.

11. roc analysis.mp.

12. roc area.mp.

13. roc auc.mp.

14. roc characteristics.mp.

15. roc curve*.mp.

16. roc curve method.mp.

17. roc estimated.mp.

18. roc evaluation.mp.

19. likelihood ratio.mp.

20. diagnostic procedure.mp.

21. Low Back Pain/di

22. diagnos*. ti,ab

23. Pain/di

24. prognosis.mp.

25. 1 or 2 or 3 or 4 or 5 or 6 or 7 or 8 or 9 or 10 or 11or 12 or 13 or 14 or 15 or 16 or 17 or 18 or 19 or 20 or 21 or 22 or 23 or 24

26. medical history taking.mp.

27. physical examination.mp.

28. Physical Examination.mp. or exp Physical Examination/

29. exp Radiography or radiography.mp

30. x-ray.mp. or exp X-ray/

31. Low Back Pain/

32. Back Pain/

33. Spine/

34. Spinal Diseases/

35. Lumbar Vertebrae/

36. magnetic resonance imaging.mp. or exp Magnetic Imaging/

37. exp Tomography, X-ray Computed/ or computed tomography.mp.

38. nuclear medicine.mp. or exp Nuclear Medicine/

39. single photon emission computed tomography.mp. or exp Tomography, Emission-Computed, Single-Photon/

40. radionuclide imaging.mp. or exp Radionuclide Imaging/

41. scintigraphy.mp.

42. bone scan.mp.

43. questionnaires.mp. or exp Questionnaires/

44. clinical history.mp.

45. diagnostic test.mp. or Diagnostic Tests, Routine/

46. thermography.mp.

47. pain provocation test*.mp.

48. 26 or 27 or 28 or 29 or 30 or 31 or 32 or 33 or 34 or 35 or 36 or 37or 38 or 39 or 40 or 41 or 42 or 43 or 44 or 45 or 46 or 47

49. spondylolisthesis/

50. spondylosis/

51. anterolisthesis.mp.

52. posterolisthesis.mp.

53. spine instability/

54. 49 or 50 or 51 or 52 or 53

55. 25 and 48

56. 54 and 55

Filters: Publication date from 2010/03/01 to 2015/05/31

Additional file 2c. CINAHL search strategy for spondylolisthesis.

| 1. | "sensitivity" AND "specificity" |
| --- | --- |
| 2. | "specificity" |
| 3. | "screening" |
| 4. | "false negative" |
| 5. | "false positive" |
| 6. | "accuracy" |
| 7. | "predictive value" |
| 8. | "predictive value of tests" |
| 9. | "reference value" |
| 10. | "roc" |
| 11. | "roc analysis" |
| 12. | "roc area" |
| 13. | "roc auc" |
| 14. | "roc characteristics" |
| 15. | "roc curve" |
| 16. | "roc curve method" |
| 17. | "roc curves" |
| 18. | "roc estimated" |
| 19. | "roc evaluation" |
| 20. | "likelihood ratio" |
| 21. | "diagnostic procedure" |
| 22. | (MH "Low Back Pain/DI") |
| 23. | "diagnos*" |
| 24. | (MH "Pain/DI") |
| 25. | "prognosis" |
| 26. | (1 OR 2 OR 3 OR 4 OR 5 OR 6 OR 7 OR 8 OR 9 OR 10 OR 11 OR 12 OR 13 OR 14 OR 15 OR 16 OR 17 OR 18 OR 19 OR 20 OR 21 OR 22 OR 23 OR 24 OR 25) |
| 27. | "medical history taking" |
| 28. | (MH "Physical Examination+") OR "physical examination" |
| 29. | (MH "Radiography+") OR "radiography" |
| 30. | (MH "X-Rays") OR "x-ray" |
|  |  |
|  |  |
| 31. | (MH "Low Back Pain/RA") |
| 32. | (MH "Back Pain/RA") |
| 33. | (MH "Spine/RA") |
| 34. | (MH "Spinal Diseases/RA") |
| 35. | (MH "Lumbar Vertebrae/RA") |
| 36. | (MH "Magnetic Resonance Imaging+") OR "magnetic resonance imaging" |
| 37. | (MH "Tomography, X-Ray Computed+") OR "computed tomography" |
| 38. | (MH "Nuclear Medicine") OR "nuclear medicine" |
| 39. | (MH "Tomography, Emission-Computed, Single-Photon+") OR "single photon emission computed tomography" |
| 40. | (MH "Back Pain") |
| 41. | (MH "Low Back Pain") |
| 42. | (MH "Spine") |
| 43. | (MH "Spinal Diseases") |
| 44. | (MH "Lumbar Vertebrae") |
| 45. | (MH "Radionuclide Imaging+") OR "radionuclide imaging" |
| 46. | "scintigraphy" |
| 47. | "bone scan" |
| 48. | (MH "Questionnaires+") OR "questionnaires" |
| 49. | "clinical history" |
| 50. | (MH "Injections, Intraspinal") |
| 51. | (MH "Diagnostic Tests, Routine") OR "diagnostic tests" |
| 52. | "thermography" |
| 53. | "pain provocation test*" |
| 54. | (S27 OR 28 OR 29 OR 30 OR 31 OR 32 OR 33 OR 34 OR 35 OR 36 OR 37 OR 38 OR 39 OR 40 OR 41 OR 42 OR 43 OR 44 OR 45 OR 46 OR 47 OR 48 OR 49 OR 50 OR 51 OR 52 OR 53) |
| 55. | spondylolisthesis |
| 56. | spondylosis |
| 57. | anterolisthesis |
| 58. | posterolisthesis |
| 59. | "spin* instability" |
| 60. | “vertebral instability” |
| 61. | (55 OR 56 OR 57 OR 58 OR 59 OR 60) |
| 62. | (26 AND 54) |
| 63. | (61 AND 62) |

Filters: Publication date from 2010/03/01 to 2015/05/31
