# Supplementary material for: Clinical classification in low back pain: best-evidence diagnostic rules based on systematic reviews
Source: BMC Musculoskelet Disord. 2017 May 12;18:188. doi: 10.1186/s12891-017-1549-6 (PMC5429540; doi:10.1186/s12891-017-1549-6)
Supplement: Supplementary file 3 — Search strategy for myofascial pain. (DOCX 19 kb) [file 12891_2017_1549_MOESM3_ESM.docx]

Additional file 3a. PubMed search strategy for myofascial pain.

1. sensitivity[MeSH Terms] AND specificity[MeSH Terms]

2. screening[Title/Abstract]

3. accuracy[Title/Abstract]

4. predictive value[Mesh Term]

5. predictive value of tests[Mesh Term]

6. reference value [Mesh Term]

7. diagnostic tests[Mesh Term]

8. low back pain[Mesh Term]

9. diagnos*[Mesh Term]

10. pain[Mesh Term]

11. prognosis[Mesh Term]

12. 1 or 2 or 3 or 4 or 5 or 6 or 7 or 8 or 9 or 10 or 11

13. medical history taking[Mesh Term]

14. physical examination[Mesh Term]

15. diagnostic test*[Mesh Term]

16. Diagnostic Tests, Routine[Mesh Term]

17. pain provocation[Title/Abstract]

18. classification[Mesh Term]

19. palpation[Title/Abstract]

20. 12 or 13 or 14 or 15 or 16 or 17 or 18 or 19

21. myofascial trigger point pain[MeSH Term]

22. myofascial pain[Title/Abstract]

23. trigger point*[Title/Abstract]

24. 21 and 22 and 23

25. 12 and 20

26. 24 and 25

Additional file 3b. EMBASE search strategy for myofascial pain.

1. (sensitivity AND specificity).mp.

2. specificity.mp.

3. accuracy .mp.

4. screening.mp.

5. false negative.mp.

6. false positive.mp.

7. predictive value.mp.

8. predictive value of tests.mp.

9. reference value.mp.

10. diagnostic procedure.mp.

11. Low Back Pain/di

12. diagnos*.mp

13. Pain/di

14. prognosis.mp.

15. 1 or 2 or 3 or 4 or 5 or 6 or 7 or 8 or 9 or 10 or 11or 12 or 13 or 14

16. medical history taking.mp.

17. Physical Examination.mp. or exp Physical Examination/

18. Low Back Pain/

19. Back Pain/

20. Spine/

21. Spinal Diseases/

22. Lumbar Vertebrae/

23. clinical history.mp.

24. diagnostic test.mp. or Diagnostic Tests, Routine/

25. pain provocation test*.mp.

26. clinical classification/

27.palpation/

28. 16 or 17 or 18 or 19 or 20 or 21 or 22 or 23 or 24 or 25 or 26 or 27

30. myofascial pain.mp

31. trigger point/

32. “*trigger point”.ab

33. 30 or 31 or 32

34. 15 and 28

35. 33 and 34

Additional file 3c. CINAHL search strategy for myofascial pain.

| 1. | "sensitivity" AND "specificity" |
| --- | --- |
| 2. | "specificity" |
| 3. | "screening" |
| 4. | "false negative" |
| 5. | "false positive" |
| 6. | "accuracy" |
| 7. | "predictive value" |
| 8. | "predictive value of tests" |
| 9. | "reference value" |
| 10. | "diagnostic procedure" |
| 11. | "diagnos*" |
| 12. | (MH "Pain/DI") |
| 13. | "prognosis" |
| 14. | (1 OR 2 OR 3 OR 4 OR 5 OR 6 OR 7 OR 8 OR 9 OR 10 OR 11 OR 12 OR 13) |
| 15. | "medical history taking" |
| 16. | (MH "Physical Examination+") OR "physical examination" |
|  |  |
| 17. | (MH "Low Back Pain/RA") |
| 18. | (MH "Back Pain/RA") |
| 19. | (MH "Spine/RA") |
| 20. | (MH "Spinal Diseases/RA") |
| 21. | (MH "Back Pain") |
| 22. | (MH "Low Back Pain") |
| 23. | (MH "Spine") |
| 24. | (MH "Spinal Diseases”) |
| 25. | "clinical history" |
| 26. | (MH "Diagnostic Tests, Routine") OR "diagnostic tests" |
| 27. | "pain provocation test*" |
| 28. | "classification" |
| 29. | "palpation" |
| 30. | (15 OR 16 OR 17 OR 18 OR 19 OR 20 OR 21 OR 22 OR 23 OR 24 OR 25 OR 26 OR  27 OR 28 OR 29) |
| 31. | (MM "Myofascial Pain Syndromes") |
| 32. | (MM "Trigger Point") |
| 33. | AB trigger point* |
| 34. | (31 OR 32 OR 33) |
| 35. | (14 AND 30) |
| 36. | (34 AND 35) |
