# Supplementary material for: Clinical classification in low back pain: best-evidence diagnostic rules based on systematic reviews
Source: BMC Musculoskelet Disord. 2017 May 12;18:188. doi: 10.1186/s12891-017-1549-6 (PMC5429540; doi:10.1186/s12891-017-1549-6)
Supplement: Supplementary file 4 — Search strategy for peripheral nerve pain. (DOCX 22 kb) [file 12891_2017_1549_MOESM4_ESM.docx]

Additional file 4a. PubMed search strategy for peripheral nerve pain.

1. sensitivity and specificity[MeSH Term]

2. screening[Title/Abstract]

3. accuracy[Title/Abstract]

4. predictive value[Mesh Term]

5. predictive value of tests[Mesh Term]

6. reference value [Mesh Term]

7. diagnostic tests[Mesh Term]

8. low back pain[Mesh Term]

9. diagnos*[Mesh Term]

10. pain[Mesh Term]

11. prognosis[Mesh Term]

12. roc curve*[MeSH Term]

13. reliability[Title/Abstract]

14. reproducibility of findings[MeSH Term]

15. 1 or 2 or 3 or 4 or 5 or 6 or 7 or 8 or 9 or 10 or 11or 12 or 13 or 14

16. medical history taking[Mesh Term]

17. physical examination[Mesh Term]

18. clinical history[Title/Abstract]

19. diagnostic test*[Mesh Term]

20. Diagnostic Tests, Routine[Mesh Term]

21. pain provocation[Title/Abstract]

22. classification[Mesh Term]

23. palpation[Title/Abstract]

24. 16 or 17 or 18 or 19 0r 20 or 21 or 22 or 23

25. neural tension[Title/Abstract]

26. myofascial pain[Title/Abstract]

27. neurodynamic*[Title/Abstract]

28. neural mobil*[Title/Abstract]

29. sciatic nerve*[MeSH Term]

30. sciatica[MeSH Term]

31. 25 and 26 and 27 and 28 and 29 and 30

32. 15 and 24

33. 31 and 32

Additional file 4b. EMBASE search strategy for peripheral nerve pain.

1. (sensitivity AND specificity).mp.

2. specificity.mp.

3. accuracy .mp.

4. screening.mp.

5. false negative.mp.

6. false positive.mp.

7. predictive value.mp.

8. predictive value of tests.mp.

9. reference value.mp.

10. diagnostic procedure.mp.

11. Low Back Pain/di

12. diagnos*.mp

13. Pain/di

14. prognosis.mp.

15. roc curve.mp

16. reliability.mp.

17. reproducibility.mp.

18. 1 or 2 or 3 or 4 or 5 or 6 or 7 or 8 or 9 or 10 or 11or 12 or 13 or 14 or 15 or 16 or 17

19. medical history taking.mp.

20. Physical Examination.mp. or exp Physical Examination/

21. Low Back Pain/

22. Back Pain/

23. Spine/

24. Spinal Diseases/

25. Lumbar Vertebrae/

26. clinical history.mp.

27. diagnostic test.mp. or Diagnostic Tests, Routine/

28. pain provocation test*.mp.

29. clinical classification/

30. palpation/

31. 19 or 20 or 21 or 22 or 23 or 24 or 25 or 26 or 27 or 28 or 29 or 30

32. sciatica.mp. or sciatica/

33. sciatic nerve.mp.

34. "neural mobil* ".ti.ab.

35. "neurodynamic*".ti.ab.

36. "neural tension".ti.ab.

37. "nerve tension".ti.ab.

38. "Straight leg raise".ti.ab.

39. "Slump test".ti.ab.

40. 32 or 33 or 34 or 35 or 36 or 37 or 38 or 39

41. 18 and 31

42. 40 and 41

Additional file 4c. CINAHL search strategy for peripheral nerve pain.

| 1. | | "sensitivity" AND "specificity" |
| --- | --- | --- |
| 2. | | "specificity" |
| 3. | | "screening" |
| 4. | | "false negative" |
| 5. | | "false positive" |
| 6. | | "accuracy" |
| 7. | | "predictive value" |
| 8. | | "predictive value of tests" |
| 9. | | "reference value" |
| 10. | | "diagnostic procedure" |
| 11. | | "diagnos*" |
| 12. | | (MH "Pain/DI") |
| 13. | | "prognosis" |
| 14. | | (MM "Roc Curve") |
| 15. | | (MM "Reproducibility of results") |
| 16. | | (MM "Reliability") |
| 17. | | (1 OR 2 OR 3 OR 4 OR 5 OR 6 OR 7 OR 8 OR 9 OR 10 OR 11 OR 12 OR 13 OR 14 OR15 OR 16) |
| 18. | | "medical history taking" |
| 19. | | (MH "Physical Examination+") OR "physical examination" |
|  | |  |
| 20. | | (MH "Low Back Pain/RA") |
| 21. | | (MH "Back Pain/RA") |
| 22. | | (MH "Spine/RA") |
| 23. | | (MH "Spinal Diseases/RA") |
| 24. | | (MH "Back Pain") |
| 25. | | (MH "Low Back Pain") |
| 26. | | (MH "Spine") |
| 27. | | (MH "Spinal Diseases”) |
| 28. | | "clinical history" |
| 29. | | (MH "Diagnostic Tests, Routine") OR "diagnostic tests" |
| 30. | | "pain provocation test*" |
| 31. | | "classification" |
| 32. | | "palpation" |
| 33. | | (18 OR 19 OR 20 OR 21 OR 22 OR 23 OR 24 OR 25 OR 26 OR  27 OR 28 OR 29 OR 30 OR 31 OR 32) |
| 34. | | (MM "Sciatica") |
| 35. | | (MM "Sciatic Nerve") |
| 36. | | "nerve tension" |
| 37. | | "neural tension" |
| 38. | | "neurodynamic*" |
| 39. | | "neuropathodynamic*" |
| 40. | | "neural mobil*" |
| 41. | | "straight leg raise" |
| 42. | | "slump test" |
| 43. | | (34 OR 35 OR 36 OR 37 OR 38 OR 39 OR 40 OR 41 OR 42) |
| 44. | | (17 AND 33) |
| 45. | | (43 AND 44) |
|  | |  |
|  | |  |
|  | |  |
