# Supplementary material for: Clinical classification in low back pain: best-evidence diagnostic rules based on systematic reviews
Source: BMC Musculoskelet Disord. 2017 May 12;18:188. doi: 10.1186/s12891-017-1549-6 (PMC5429540; doi:10.1186/s12891-017-1549-6)
Supplement: Supplementary file 5 — Characteristics of the included studies. (DOCX 37 kb) [file 12891_2017_1549_MOESM5_ESM.docx]

Additional file 5. Characteristics of the included studies.

| Diagnostic categories | N | N on whom results are based | Mean age  (Range) | % Male | Characteristics of participants | Setting and design | Type of disorder | Reference standard | Index test |
| --- | --- | --- | --- | --- | --- | --- | --- | --- | --- |
| **Intervertebral disc** | | | | | | | | | |
| Donelson 1997 [39] | 63 | 63 | 40 (?) | 65 | Patients with chronic LBP +/- leg pain, referred for discography | Setting: secondary  Design: prospective | Disc | Discography with control disc | Physical examination |
| Laslett 2005 [41] | 83 | 69 | 43 (?) | 55 | Patients with chronic LBP +/- leg pain, referred for diagnostic injections | Setting: secondary  Design: prospective | Disc | Discography with control disc | Physical examination |
| Young 2003 [40] | 24 | 24 | 41 (18-79) | 39 | Patients with chronic LBP +/- leg pain, referred for diagnostic injections | Setting: secondary  Design: prospective | Disc | Discography with control disc | Physical examination and history items |
| Schwarzer 1995 [42] | 36 | 32 | 37 (31-43) | 66 | Patients with chronic LBP and positive discography | Setting: secondary  Design: prospective | Disc | Discography with control disc | History items |
| **Facet joint** | | | | | | | | | |
| Manchikanti 2000 [43] | 200 | 200 | 47 (?) | 40 | Patients with chronic LBP +/- leg pain, referred for pain mangement | Setting: secondary  Design: prospective | Facet joint | Confirmatory nerve block > 75% relief | Physical examination and history items |
| Manchikanti 1999 [44] | 81 | 81 | 47 (?) | 42 | Patients with chronic LBP +/- leg pain, referred for pain mangement | Setting: secondary  Design: prospective | Facet joint | Confirmatory nerve block > 75% relief | Physical examination and history items |
| Manchikanti 2008 [49] | 303 | 303 | 47 (?) | 42 | Patients with chronic LBP +/- leg pain, referred for pain mangement | Setting: secondary  Design: retrospective | Facet joint | Confirmatory nerve block > 75% relief | Physical examination and history items |
| Schwarzer 1994 [50] | 164 | 164 | 38 (?) | 60 | Patients with chronic LBP +/- leg pain, referred for diagnostic injections | Setting: secondary  Design: prospective | Facet joint | Confirmatory intra-articular or nerve block > 50% relief | Physical examination and history items |
| Fairbank 1981 [51] | 25 | 25 | 35 (19-30) | 70 | Patients with chronic LBP +/- leg pain, referred for diagnostic injections | Setting: tertiary  Design: prospective | Facet joint | Controlled intra-articular block ?% relief | Physical examination |
| Young 2003 [40] | 24 | 24 | 41 (18-79) | 40 | Patients with chronic LBP +/- leg pain, referred for diagnostic injections | Setting: secondary  Design: prospective | Facet joint | Single intra-articular block > 80% relief | Physical examination and history items |
| Laslett 2003, 2006 [47] | 120 | 92 | 43 (?) | 54 | Patients with chronic LBP +/- leg pain, signs of FJ pain based on MRI, referred for diagnostic injections | Setting: secondary  Design: prospective | Facet joint | Single intra-articular or nerve block block > 95% relief/>75% relief | Physical examination |
| Revel 1998 [46] | 42 | 42 | 59 (?) | 33 | Patients with chronic LBP +/- leg pain, referred for FJ injections | Setting: tertiary  Design: prospective | Facet joint | Single intra-articular block > 75% relief | Physical examination and history items |
| Revel 1992 [45] | 40 | 40 | 64 (?) | 35 | Patients with chronic LBP +/- leg pain, referred for FJ injections | Setting: tertiary  Design: prospective | Facet joint | Single intra-articular block > 75% relief | Physical examination and history items |
| **Sacroiliac joint** | | | | | | | | | |
| Laslett 2003 [52] | 43/34 | 43/34 | 42 (20-79) | Unknown | Patients with chronic LBP below L5, +/- leg pain, referred for diagnostic injections | Setting: secondary  Design: prospective | Sacroiliac joint | Confirmatory intra-articular block > 80% relief | Physical examination |
| Van der Wurff 2006 [53], 2006 [57] | 60 | 60 | 51 (?) | 13 | Patients with chronic LBP below L5, over the SIJ, +/- leg pain, referred for diagnostic injections | Setting: tertiary  Design: prospective | Sacroiliac joint | Comparative dual intra-articular block > 50% relief | Physical examination |
| Stanford 2010 [55] | 34 | 34 | Unknown | Unknown | Patients with chronic LBP below L5, over the SIJ, +/- leg pain, failed noninvasive treatment | Setting: tertiary  Design: prospective | Sacroiliac joint | Confirmatory intra-articular block (30 min.s apart) > 79% relief | Physical examination |
| Ozgocmen 2008 [56] | 40 | 40 | 26 (?) | 60 | Patients with chronic LBP and suspicion of spondyloarthritis | Setting: tertiary  Design: prospective | Sacroiliitis | Magnetic resonance imaging (MRI) | Physical examination |
| Dreyfuss 1996 [54] | 85 | 85 | 45 (18-87) | 28 | Patients with chronic LBP below L5, over the SIJ, +/- leg pain, referred for SIJ injections | Setting: tertiary  Design: prospective | Sacroiliac joint | Single intra-articular block> 80% relief |  |
| Young 2003 [40] | 24 | 24 | 41 (18-79) | 40 | Patients with chronic LBP +/- leg pain, referred for diagnostic injections | Setting: secondary  Design: prospective | Sacroiliac joint | Single intra-articular block | Physical examination and history items |
| **Disc herniation with nerve root involvement** | | | | | | | | | |
| Hancock 2011 [60] | 283 | 283 | 42 (18-65) | 66 | Patients with subacute sciatica, clinical signs of NRI, and confirmed disc herniation any level | Setting: tertiary  Design: prospective | Disc herniation | MRI | Physical examination and history items |
| Albeck 1996 [69] | 80 | 61 | 40 (21-59) | 60 | Patients with persistent monoradicular pain corresponding L5-S1 nerves +/- one neurological sign | Setting: tertiary  Design: prospective | Nerve root | Surgical findings | Physical examination and history items |
| Suri 2010 [64] | 54 | 54 | 55 | 87 | Patients with subacute monoradicular pain any level | Setting: tertiary  Design: prospective | Nerve root | MRI | Physical examination |
| Gurdjian 1961 [65] | 1176 | 1176 | Unknown | 66 | Patients with persistent radicular pain any level | Setting: secondary  Design: prospective | Nerve root | Disc protusions on MRI | Physical examination and history items |
| Kerr 1988 [63] | 136 | 136 | Unknown | 55 | Patients with persistent sciatica any level | Setting: tertiary  Design: retrospective | Disc herniation or protusion | Disc protusions on Myologram | Physical examination |
| Knutsson 1961 [66] | 182 | 182 | Unknown | 60 | Patients with confirmed disc herniation any level and clinical signs of NRI | Setting: tertiary  Design: retrospective | Disc herniation or protusion | Surgical findings | Physical examination |
| Spangfort 1972 [70] | 2377 | 2152 | 41 (15-74) | 70 | Patients with confirmed disc herniation any level and clinical signs of NRI | Setting: tertiary  Design: prospective | Disc herniation or protusion | Surgical findings | Physical examination |
| Charnley 1951 [80] | 88 | 88 | Unknown | Unknown | Patients with confirmed disc herniation any level and clinical signs of NRI | Setting: tertiary  Design: prospective | Disc herniation or protusion | Surgical findings | Physical examination |
| Demircan 2002 [79] | 284 |  | 32 (20-45) | 51 | Patients with confirmed L4-L5 disc herniations and clinical signs of NRI | Setting: tertiary  Design: prospective | Disc herniation or protusion | Surgical findings | Physical examination |
| Poiraudeau 2001 [77] | 78 | 77 | 50 (?) | 42 | Patients with confirmed disc herniation any level, radicular pain in dermatomes L5-S1, and clinical signs of NRI | Setting: tertiary  Design: prospective | Disc herniation or protusion | Herniations on MRI | Physical examination |
| Kosteljanetz 1984 [71] | 100 | 100 | Unknown | 51 | Patients with sciatica or clinical signs of NRI | Setting: tertiary  Design: prospective | Disc herniation or protusion | Surgical findings | Physical examination |
| Kosteljanetz 1988 [78] | 52 | 52 | 45 (18-73) | 60 | Patients with sciatica or clinical signs of NRI | Setting: tertiary  Design: prospective | Disc herniation or protusion | Surgical findings | Physical examination |
| Vucetic 1996 [68] | 163 | 163 | 43 (19-68) | 53 | Patients with sciatica or clinical signs of NRI | Setting: tertiary  Design: prospective | Disc herniation or protusion | Surgical findings | Physical examination |
| Hakelius 1972 [72] | 1959 | 1959 | Unknown | Unknown | Patients with sciatica suspected of NRI | Setting: tertiary  Design: prospective | Disc herniation or protusion | Surgical findings | Physical examination |
| Stankovic 1999 [67] | 105 | 105 | 43 (19-64) | 66 | Patients with low back pain and/or sciatica considered for surgery | Setting: tertiary  Design: prospective | Disc herniation + nerve root compromise | MRI | Physical examination |
| Vroomen 1998 [82] | 71 | 71 | 39 (?) | 65 | Patients with monoradicular pain any level considered for surgery | Setting: tertiary  Design: prospective | Nerve root | MRI | Physical examination |
| Vroomen 2002 [61] | 274 |  | 46 (?) | 51 | Patients with persistent sciatica | Setting: primary  Design: prospective | Nerve root | MRI | Physical examination |
| Bertilson 2010 [62] | 61 | 61 | 60 (27-80) | 51 | Patients with LBP +/- leg pain referred for radiographs | Setting: secondary  Design: prospective | Nerve root | MRI | Physical examination and history items |
| Majlesi 2008 [81] | 75 | 75 | 40 (?) | 34 | Patients with subacute LBP or leg pain | Setting: tertiary  Design: prospective | Disc herniation or protusion | MRI | Physical examination |
| Haldeman 1988 [76] | 100 | 99 | ? (21-67) | Unknown | Patients with persistent sciatica referred for radiographs not considered for surgery | Setting: primary  Design: prospective | Disc herniation or protusion + spinal stenosis | CT-scan | Physical examination |
| Meylemans 1988* [75] | 146 | 146 | ? (13-77) | 58 | Patients with persistent sciatica referred for radiographs | Setting: secondary  Design: prospective | Disc herniation or protusion | CT-scan | Physical examination |
| **Spinal Stenosis** | | | | | | | | | |
| Konno 2007 [84]  Derivation 1 | 250 | 234 | 63 (?) | 48 | Consecutive patients with primary symptoms of pain and numbness in the legs. All had surgery. | Setting: tertiary  Design: unclear | Any type of spinal stenosis | Consensus by experts based on physical examination, X-ray, MRI, and surgery | Derivation 1: questionnaire items |
| Sugiaka 2008 [89] | 468 | 374 | Unknown | Unknown | Consecutive patients with primary symptoms of pain and numbness in the legs. | Setting: secondary and tertiary  Design: unclear | Any type of spinal stenosis | Consensus by experts based on physical examination, X-ray and MRI | Questionnaire items |
| Dong 1989 [92] | 30 | A.19  B. 11 | 59 (38-71) | 90 | Patients with LBP and walking tolerance < 400 m  A.confirmed spinal stenosis  B.confirmed intermittant claudication | Setting: tertiary  Design: retrospective | Spinal stenosis not specified | A. myelography and CT-scan  B. arteriography | Treadmill test and questionnaire items |
| Katz 1995 [86] | 93 | 75 | 65 (40-91) | 31 | Patients with LBP with or without leg pain | Setting: tertiary  Design: prospective | Narrowing of the spinal canal or neural foramina | >80% confidence in expert opinion based on physical examination and MRI in 88% of LSS cases | Physical examination and history items |
| Fritz 1997 [90] | 45 | 45 | 58 (?) | Unknown | Patients with LBP and leg pain who reported limited walking tolerance | Setting: tertiary  Design: prospective | Any type of spinal stenosis | MRI and CT-scan | Treadmill test and questionnaire items |
| Jensen 1989 [87] | 23 | 23 | 55 (23-72) | 57 | Patients with neurogenic claudication and indication for myelography | Setting: unknown  Design: prospective | Spinal stenosis not specified | Myelography | Treadmill test |
| Cook 2011 [83] | 1448 | 1448 | 55 (?) | 41 | Consecutive patients suspected to have spinal stenosis | Setting: tertiary  Design: prospective | Any type of spinal stenosis | Expert opinion based on physical examination and MRI | Physical examination and history items |
| Ljunggren 1991 [85] | 179 | 179 | Unknown | 58 | Consecutive patients with LBP and leg pain. Potential surgery candidates. Confirmed spinal stenosis or disc herniation | Setting: secondary  Design: unclear | Canal or recess stenosis | Physical examination, imaging and surgery | McGill questionnaire items |
| Roach 1997 [88] | 106 | 99 | 55 (19-68) | 51 | Patients with recurrent or chronic LBP. Disc disease with spinal stenosis. | Setting: tertiary  Design: prospective | Narrowing of the spinal canal or neural foramina | Physician opinion based on physical examination and imaging. | Physical examination and history items |
| **Spondylolisthesis** | | | | | | | | | |
| Fritz 2005 [94] | 49 | 49 | 39 (?) | 43 | Patients with subacute LBP suspected to have segmental instability | Setting: tertiary  Design: prospective | Spondylolisthesis not specified. Rotational or translational instability | Flexion-extension radiographs | Physical examination |
| Abbott 2005 [95] | 123 | 123 | 40 (20-75) | 55 | Patients with persistent LBP | Setting: primary  Design: prospective | Rotational or translational instability | Flexion-extension radiographs | Physical examination |
| Kasai 2006 [96] | 122 | 122 | 69 (39-88) | 52 | Elderly patients with persistent LBP and suspected stenosis, scoliosis, or spondylolisthesis | Setting: tertiary  Design: prospective | Degenerative spondylolisthesis. Angular or translational instability | Flexion-extension radiographs | Physical examination |
| Ferrari 2014 [99] | 119 | 102 | 45 (?) | 44 | Patients with subacute LBP suspected to have segmental instability | Setting: tertiary  Design: prospective | Degenerative or isthmic spondylolisthesis. Rotational or translational instability | Flexion-extension radiographs | Physical examination |
| Ahn 2015 [100] | 96/73 | 96/73 | 55 (?) | 30 | Patients with subacute LBP +/- leg pain referred for radiographs suspected to have segmental instability | Setting: tertiary  Design: prospective | Degenerative or spondylotic spondylolisthesis. Rotational or translational instability | Lateral radiographs and flexion-extension radiographs | Physical examination |
| Kalpakcioglu 2009 [97] | 130 | 130 | 55 (22-78) | 13 | Patients with chronic LBP +/- leg pain referred for radiographs. | Setting: tertiary  Design: prospective | Spondylolisthesis not specified. Translational instability | Flexion-extension radiographs | Physical examination  and history items |
| Collear 2006 [98] | 44 | 44 | 40 (18-80) | 48 | Patients with chronic LBP +/- radiculopathy referred for radiographs | Setting: tertiary  Design: prospective | Isthmic spondylolisthesis. Translational instability | Lateral static radiographs | Physical examination |
| Sundell 2013 [101] | 25 | 25 | 15 (13-19) | 56 | Teenager athletes with acute LBP +/- radiculopathy referred for radiographs | Setting: tertiary  Design: prospective | Isthmic spondylolisthesis with stress fracture | MRI and CT-scan | Physical examination |
| **Fracture** | | | | | | | | | |
| Henschke 2009 [103] | 1172 | 1172 | 44 (?) | 53 | Patients with acute LBP +/- leg pain who presented to a primary care provider | Setting: primary  Design: prospective | Fracture | Health care provider judgment | History items |
| van den Bosch 2004 [104] | 2007 | 2007 | 53 (?) | 42 | Patients with acute LBP +/- leg pain referred for radiographs | Setting: tertiary  Design: retrrospective | Fracture | Radiographs | History items |
| Roman 2010 [109] | 1448 | 1448 | Unknown | 41 | Patients with acute LBP +/- leg pain referred for radiographs | Setting: tertiary  Design: retrospective | Osteoporotic fracture | Radiographs or CT-scan | Physical examination  and history items |
| Deyo 1986 [107] | 621 | 311 | 41 (15-86) | 47 | Patients with LBP +/- leg pain referred for radiographs | Setting: primary  Design: prospective | Fracture | Radiographs | History items |
| Gibson 1992 [105] | 225 | 108 | ? (16-65) | Unknown | Patients with acute LBP following trauma +/- leg pain referred for radiographs | Setting: tertiary  Design: prospective | Fracture | Radiographs | Physical examination  and history items |
| Patrick 1983 [106] | 552 | 552 | ? (6-95) | 53 | Patients with LBP +/- leg pain referred for radiographs | Setting: tertiary  Design: retrospective | Fracture | Radiographs | Physical examination  and history items |
| Reinus 1998 [108] | 482 | 482 | 56 (17-98) | 35 | Patients with LBP +/- leg pain referred for radiographs | Setting: tertiary  Design: prospective | Fracture | Radiographs | Physical examination  and history items |
| Scavone 1981* [110] | 871 | 871 | Unknown | Unknown | Patients with LBP +/- leg pain and radiographs taken | Setting: tertiary  Design: retrospective | Fracture | Radiographs | Physical examination  and history items |

*Description transferred from previous systematic reviews. ? = No original data presented to allow for calculation.
