# Supplementary material for: Clinical classification in low back pain: best-evidence diagnostic rules based on systematic reviews
Source: BMC Musculoskelet Disord. 2017 May 12;18:188. doi: 10.1186/s12891-017-1549-6 (PMC5429540; doi:10.1186/s12891-017-1549-6)
Supplement: Supplementary file 6 — Quality assessment of included studies. (DOCX 35 kb) [file 12891_2017_1549_MOESM6_ESM.docx]

Additional file 6. Quality assessment of included studies

|  | **1** | **2** | **3** | **4** | **5** | **6** | **7** | **8** | **9** | **10** | **11** | **12** | **13** | **14** |
| --- | --- | --- | --- | --- | --- | --- | --- | --- | --- | --- | --- | --- | --- | --- |
| **Disc** | | | | | | | | | | | | | | |
| Donelson 1997 [39] | N | Y | Y | Y | Y | Y | Y | Y | Y | Y | Y | Y | N | Y |
| Laslett 2005* [41] | N | Y | Y | U | Y | Y | Y | Y | Y | Y | Y | Y | N | Y |
| Young 2003* [40] | N | U | Y | U | N | Y | Y | Y | Y | Y | Y | Y | N | U |
| Schwarzer 1995[42] | N | Y | Y | U | Y | Y | Y | Y | Y | U | U | Y | Y | N |
| **Facet joint** | | | | | | | | | | | | | | |
| Manchikanti  2000 [43] | N | Y | Y | U | Y | Y | Y | U | Y | U | U | Y | N | Y |
| Manchikanti  1999 [44] | N | Y | Y | U | Y | Y | Y | U | Y | U | U | U | N | Y |
| Manchikanti 2008 [49] | Y | Y | Y | Y | Y | Y | Y | Y | Y | Y | U | Y | Y | Y |
| Schwarzer 1994[50] | N | Y | Y | Y | Y | Y | Y | Y | Y | U | U | Y | Y | Y |
| Laslett 2006* [47] | N | Y | Y | Y | Y | N | Y | Y | Y | Y | Y | Y | Y | Y |
| Fairbank 1981 [51] | Y | Y | Y | Y | Y | Y | Y | Y | Y | U | Y | Y | Y | Y |
| Revel 1998 [46] | N | Y | Y | U | Y | Y | Y | U | Y | Y | Y | U | N | Y |
| Revel 1992 [45] | Y | Y | Y | Y | Y | Y | Y | U | Y | U | U | U | N | Y |
| **Sacroiliac joint** | | | | | | | | | | | | | | |
| Laslett 2003* [52] | N | Y | Y | Y | N | Y | Y | Y | Y | Y | Y | Y | N | Y |
| van der Wurff 2006 [53], 2006 [57] | N | Y | Y | Y | Y | Y | Y | Y | Y | Y | Y | U | N | Y |
| Ozgocmen 2008 [56] | N | Y | Y | Y | Y | Y | Y | Y | Y | Y | Y | Y | Y | Y |
| Stanford 2010 [55] | U | Y | Y | Y | Y | Y | Y | Y | Y | Y | U | Y | Y | Y |
| Dreyfuss 1996 [54] | Y | Y | Y | U | Y | Y | Y | Y | Y | Y | Y | U | Y | Y |
| **Nerve root involvement** | | | | | | | | | | | | | | |
| Hancock 2011 [60] | N | Y | Y | Y | Y | Y | Y | Y | Y | Y | Y | U | Y | Y |
| Albeck 1996 [69] | N | U | Y | U | Y | N | Y | U | U | N | Y | U | U | U |
| Suri 2010 [64] | N | Y | Y | Y | Y | Y | Y | Y | Y | Y | Y | Y | Y | Y |
| Gurdjian 1961 [65] | N | U | U | U | Y | Y | U | N | U | U | U | Y | Y | Y |
| Kerr 1988 [63] | N | Y | Y | U | Y | N | Y | U | U | U | Y | Y | U | Y |
| Knutsson 1961 [66] | N | Y | Y | U | Y | Y | U | N | N | U | U | Y | N | Y |
| Spangfort 1972 [70] | N | U | Y | U | Y | Y | U | N | U | U | U | Y | Y | Y |
| Charnley 1951 [80] | N | U | Y | U | Y | Y | U | U | U | U | U | U | Y | Y |
| Demircan 2002 [79] | N | Y | Y | U | Y | N | U | U | U | Y | U | Y | U | Y |
| Poiraudeau 2001 [77] | N | Y | Y | U | Y | N | Y | Y | Y | Y | Y | Y | Y | Y |
| Kosteljanetz 1984 [71] | N | Y | Y | U | Y | Y | Y | U | U | U | Y | U | Y | Y |
| Kosteljanetz 1988 [78] | N | Y | Y | U | N | N | Y | Y | U | U | Y | N | Y | Y |
| Vucetic 1996 [68] | N | Y | Y | U | Y | Y | Y | Y | Y | U | U | Y | Y | Y |
| Hakelius 1972 [72] | N | Y | Y | U | Y | Y | U | U | U | U | Y | Y | Y | U |
| Stankovic 1999 [67] | N | Y | Y | U | Y | Y | Y | Y | Y | Y | Y | Y | Y | Y |
| Vroomen 1998 [82] | N | U | Y | U | Y | Y | Y | N | Y | Y | Y | Y | Y | U |
| Vroomen 2002 [61] | Y | U | Y | U | Y | Y | Y | N | Y | Y | Y | Y | Y | Y |
| Bertilson 2010 [62] | N | Y | Y | Y | Y | Y | Y | N | Y | Y | Y | Y | U | Y |
| Majlesi 2008 [81] | N | U | Y | U | Y | Y | Y | U | U | Y | Y | U | U | Y |
| Haldeman 1988 [76] | Y | Y | Y | U | Y | Y | U | Y | U | U | U | Y | U | Y |
| Meylemans 1988* [75] | N | Y | Y | U | Y | Y | Y | U | U | U | U | Y | Y | Y |
| **Spinal stenosis** | | | | | | | | | | | | | | |
| Konno 2007 [91] | N | Y | Y | U | Y | Y | Y | Y | N | U | U | Y | Y | Y |
| Sugioka 2008 [89] | N | N | Y | U | Y | Y | N | Y | N | Y | U | Y | N | N |
| Dong 1989 [92] | N | N | Y | U | N | Y | Y | Y | N | N | Y | Y | Y | Y |
| Katz 1995 [86] | N | Y | N | U | Y | Y | N | N | N | Y | Y | Y | U | N |
| Fritz 1997 [90] | Y | Y | N | U | Y | N | Y | Y | N | U | Y | U | N | N |
| Jensen 1989 [87] | Y | N | N | U | Y | Y | Y | N | N | Y | Y | Y | N | U |
| Cook 2011[83] | N | N | N | U | Y | U | N | Y | N | U | N | Y | U | U |
| Ljunggren 1991[85] | Y | Y | Y | U | Y | U | Y | Y | Y | Y | U | Y | U | U |
| Roach 1997 [88] | Y | Y | Y | U | Y | N | N | U | N | Y | Y | Y | N | Y |
| **Spondylolisthesis** | | | | | | | | | | | | | | |
| Fritz 2005 [94] | N | Y | Y | Y | Y | Y | Y | N | Y | Y | Y | Y | Y | Y |
| Abbott 2005 [95] | Y | Y | Y | U | Y | Y | Y | U | Y | Y | Y | Y | Y | Y |
| Kasai 2006 [96] | N | Y | Y | U | Y | Y | Y | Y | Y | Y | Y | Y | Y | Y |
| Ferrari 2014 [99] | N | N | Y | U | Y | U | Y | Y | N | Y | Y | Y | N | N |
| Ahn 2015 [100] | N | Y | Y | N | Y | Y | Y | Y | Y | Y | Y | Y | Y | Y |
| Kalpakcioglu 2009 [97] | N | Y | U | Y | Y | Y | Y | N | Y | U | U | U | Y | Y |
| Collear 2006 [98] | N | Y | Y | Y | Y | Y | Y | Y | Y | Y | Y | Y | Y | Y |
| Sundell 2013 [101] | N | Y | Y | N | Y | Y | Y | N | N | Y | U | Y | Y | Y |
| **Fracture** | | | | | | | | | | | | | | |
| Henschke 2009 [103] | Y | Y | N | U | Y | Y | U | Y | Y | U | U | Y | Y | Y |
| van den Bosch 2004 [104] | Y | Y | Y | U | N | Y | Y | Y | N | U | Y | Y | N | N |
| Roman 2010 [109] | N | Y | Y | Y | Y | N | Y | N | U | U | U | Y | Y | Y |
| Deyo 1986 [107] | Y | Y | Y | Y | N | U | Y | Y | N | N | Y | Y | N | U |
| Gibson 1992 [105] | N | Y | Y | U | N | U | Y | Y | N | N | Y | Y | N | U |
| Patrick 1983 [106] | N | U | Y | Y | Y | Y | Y | N | N | U | U | Y | N | U |
| Reinus 1998 [108] | N | U | Y | U | U | U | Y | N | N | N | Y | Y | N | Y |
| Scavone 1981* [110] | U | N | Y | U | U | U | Y | ? | ? | U | Y | Y | N | N |

Y=Yes, N= No, U=Unclear, ?=No data available. * Transferred from assessment in recent reviews

Supplement to Additional file 2. Guide to scoring QUADAS quality assessment items

| **Item** | **Criteria and scoring** |
| --- | --- |
| **1** | **Spectrum bias. Was the spectrum of patients representative of the patients who will receive the test in practice?**  Yes: A consecutive series of patients or a random sample has been selected that are relevant for patients seen in primary care. Information should be given about setting, inclusion and exclusion criteria, and preferably number of patients eligible and excluded. If a mixed population of primary and secondary care patients is used: the number of participants from each setting is presented  No: Only patients with confirmed diagnosis at the start of the study were included. Healthy controls are used. Also, score ‘no’ if non-response is high and selective, or there is clear evidence of selective sampling.  Unclear: Insufficient information is given on the setting, selection criteria, or selection procedure to make a judgment |
| **2** | **Were selection criteria clearly described?**  Yes: All relevant information regarding how participants were selected for inclusion in the study was provided  No: Study selection criteria were not clearly reported  Unclear: Insufficient information is given on the method of recruitment and selection criteria |
| **3** | **Verification bias. Is the reference standard likely to classify the target condition correctly?**  Yes: Paraclinical findings; i.e. plain radiography, magnetic resonance imaging (MRI), computed tomography (CT), injections, or surgical findings; are used as a reference standard  No: Seriously questioning the methods used, if only consensus among observers, or an unknown combination of the clinical assessment (“clinical judgment”) is used as reference standard.  Unclear: Insufficient information is given on the reference standard to make an adequate assessment |
| **4** | **Disease progression bias. Is the period between reference standard and index test short enough to be reasonably sure that the target condition did not Change between the two tests?**  Yes: The time period between clinical assessment and the reference standard is two weeks or less  No: The time period between clinical assessment and the reference standard is longer than two weeks  Unclear: The time period between clinical assessment and the reference standard is not reported |
| **5** | **Partial verification bias. Did the whole sample or a random selection of the sample, receive verification using a reference standard of diagnosis?**  Yes: it is clear that all patients who received the index test went on to receive a reference standard, even if the reference standard is not the same for all patients  No: Not all patients who received the index test received verification by a reference standard  Unclear: Insufficient information is provided to assess this item |
| **6** | **Differential verification. Did patients receive the same reference standard regardless of the index test result?**  Yes: it is clear that all patients receiving the index test are subjected to the same reference standard  No: Different reference standards are used  Unclear: Insufficient information is provided to assess this item |
| **7** | **Incorporation bias. Was the reference standard independent of the index test (i.e. the index test did not form part of the reference standard)?**  Yes: The index test is not part of the reference standard  No: The index test is clearly part of the reference standard  Unclear: Insufficient information is provided to assess this item |
| **8** | **Was the execution of the index test described in sufficient detail to permit replication of the test?**  Yes: A sufficient description of the execution of index test (or a citation)  No: The execution of index test is not described  Unclear: The execution of index test is insufficiently described |
| **9** | **Was the execution of the reference standard described in sufficient detail to permit its replication?**  Yes: A sufficient description of the execution of reference standard (or a citation)  No: The execution of the reference standard is not described  Unclear: The execution of index test is insufficiently described |
| **10** | **Test review bias. Were the index test results interpreted without knowledge of the results of the reference standard?**  Yes: The results of the index test are interpreted blind to the results of the reference test. Also, classify as ‘yes’ if the sequence of testing is always the same (i.e. the index test is always performed first, followed by the reference standard), and consequently, the index test is interpreted blind of the reference standard  No: The assessor is aware of the results of the reference standard  Unclear: Insufficient information is given on independent or blind assessment of the reference standard |
| **11** | **Test review bias. Were the reference standard results interpreted without knowledge of the results of the index test?**  Yes: The results of the reference standard are interpreted blind to the results of the index tests. Also, classify as ‘yes’ if the sequence of testing is always the same (i.e. the reference standard is always performed first, followed by the index test) and consequently, the reference standard is interpreted blind of the index test  No: The assessor is aware of the results of the index test  Unclear: Insufficient information is given on independent or blind assessment of the index test |
| **12** | **Were the same clinical data available when test results were interpreted as would be available when the test is used in practice?**  Yes: Clinical data (i.e. patient history, other physical tests) would normally be available when the test results are interpreted and similar data are available in the study. Also, classify as ‘yes’ if clinical data would normally not be available when the test results are interpreted and these data are also not available in the study  No: This is not the case, e.g. if other test results are available that cannot be regarded as part of routine care  Unclear: The paper does not explain which clinical information was available at the time of assessment |
| **13** | **Were uninterpretable/intermediate test results reported?**  Yes: All test results are reported for all patients, including uninterpretable, indeterminate, or intermediate results. Also, classify as ‘yes’ if the authors do not report any uninterpretable, indeterminate, or intermediate results AND the results are reported for all patients who were described as having been entered into the study  No: There is suspicion that such results occurred, but have not been reported  Unclear: It is unclear whether all results have been reported |
| **14** | **Were withdrawals from the study explained?**  Yes: It is clear what happens to all patients who entered the study (all patients are accounted for, preferably in a flow chart). Also, classify as ‘yes’ if the authors do not report any withdrawals AND if the results are available for all patients who were reported to have been entered in the study  No: It is clear that not all patients who were entered completed the study (received both index test and reference standard), and not all patients are accounted for  Unclear: The paper does not clearly describe whether or not all patients completed all tests, and are included in the analysis |
|  |  |
