# Supplementary material for: Clinical classification in low back pain: best-evidence diagnostic rules based on systematic reviews
Source: BMC Musculoskelet Disord. 2017 May 12;18:188. doi: 10.1186/s12891-017-1549-6 (PMC5429540; doi:10.1186/s12891-017-1549-6)
Supplement: Supplementary file 7 — Flow chart for selection of disc, sacroiliac joint and facet joint articles. (DOCX 12 kb) [file 12891_2017_1549_MOESM7_ESM.docx]

Additional file 7. Flow chart for selection of disc, sacroiliac joint and facet joint articles

Records rejected based on title/abstract

n = 8.425

Studies excluded, did not meet all inclusion criteria n = 100

Additional studies identified through reference list searching

n = 45

Full text of potentially relevant studies retrieved

n = 61

Studies read in full text

n = 106

Final new studies included in review

Disc n = 1

Facet joint n = 2 Sacroiliac joint n = 3

Records identified through searches 2006-2015

PubMed n = 5.149

Embase n = 1.398 after dublicates removed

Cinahl n = 1.927 after dublicates removed
