# Supplementary material for: Clinical classification in low back pain: best-evidence diagnostic rules based on systematic reviews
Source: BMC Musculoskelet Disord. 2017 May 12;18:188. doi: 10.1186/s12891-017-1549-6 (PMC5429540; doi:10.1186/s12891-017-1549-6)
Supplement: Supplementary file 8 — Flow chart for selection of spondylolisthesis articles. (DOCX 12 kb) [file 12891_2017_1549_MOESM8_ESM.docx]

Additional file 8. Flow chart for selection of spondylolisthesis articles

Final new studies included in review

n = 5

Records rejected based on title/abstract

n = 1.158

Studies excluded, did not meet all inclusion criteria n = 5

Additional studies identified through reference list searching

n = 2

Full text of potentially relevant studies retrieved

n = 8

Studies read in full text

n = 10

Records identified through searches 2010-2015

PubMed n = 1.015

Embase n = 112 after dublicates removed

Cinahl n = 39 after dublicates removed
