# Supplementary material for: Clinical classification in low back pain: best-evidence diagnostic rules based on systematic reviews
Source: BMC Musculoskelet Disord. 2017 May 12;18:188. doi: 10.1186/s12891-017-1549-6 (PMC5429540; doi:10.1186/s12891-017-1549-6)
Supplement: Supplementary file 9 — Flow chart for selection of myofascial pain articles. (DOCX 12 kb) [file 12891_2017_1549_MOESM9_ESM.docx]

Additional file 9. Flow chart for selection of myofascial pain articles

Final new studies included in review

n = 0

Records rejected based on title/abstract

n = 1.415

Studies excluded, did not meet all inclusion criteria n = 16

Additional studies identified through reference list searching

n = 2

Full text of potentially relevant studies retrieved

n = 14

Studies read in full text

n = 16

Records identified through searches -2015

PubMed n = 1.257

Embase n = 85 after dublicates removed

Cinahl n = 87 after dublicates removed
