# Supplementary material for: Clinical classification in low back pain: best-evidence diagnostic rules based on systematic reviews
Source: BMC Musculoskelet Disord. 2017 May 12;18:188. doi: 10.1186/s12891-017-1549-6 (PMC5429540; doi:10.1186/s12891-017-1549-6)
Supplement: Supplementary file 10 — Flow chart for selection of nerve pain articles. (DOCX 12 kb) [file 12891_2017_1549_MOESM10_ESM.docx]

Additional studies identified through reference list searching

n = 31

Additional file 10. Flow chart for selection of peripheral nerve pain articles

Final new studies included in review

n = 0

Records rejected based on title/abstract

n = 4.079

Studies excluded, did not meet all inclusion criteria n = 58

Full text of potentially relevant studies retrieved

n = 27

Studies read in full text

n = 58

Records identified through searches -2015

PubMed n = 3.937

Embase n = 105 after dublicates removed

Cinahl n = 64 after dublicates removed
